# Identification of immune-related lncRNA in sepsis by construction of ceRNA network and integrating bioinformatic analysis

**DOI:** 10.1186/s12864-023-09535-7

**Published:** 2023-08-24

**Authors:** Tianfeng Wang, Si Xu, Lei Zhang, Tianjun Yang, Xiaoqin Fan, Chunyan Zhu, Yinzhong Wang, Fei Tong, Qing Mei, Aijun Pan

**Affiliations:** 1 Department of Critical Care Medicine, Division of Life Science and Medicine, The First Affiliated Hospital of USTC, University of Science and Technology of China, Hefei, 230001 Anhui Province China; 2 Department of Neurology, The Second Affiliated Hospital of Anhui Medical University, Hefei, China

**Keywords:** Sepsis, Immune-related genes, lncRNA, Biomarker, Network analysis

## Abstract

**Background:**

Sepsis is a high mortality disease which seriously threatens human life and health, for which the pathogenetic mechanism still unclear. There is increasing evidence showed that immune and inflammation responses are key players in the development of sepsis pathology. LncRNAs, which act as ceRNAs, have critical roles in various diseases. However, the regulatory roles of ceRNA in the immunopathogenesis of sepsis have not yet been elucidated.

**Results:**

In this study, we aimed to identify immune biomarkers associated with sepsis. We first generated a global immune-associated ceRNA (IMCE) network based on data describing interactions pairs of gene–miRNA and miRNA–lncRNA. Afterward, we excavated a dysregulated sepsis immune-associated ceRNA (SPIMC) network from the global IMCE network by means of a multi-step computational approach. Functional enrichment indicated that lncRNAs in SPIMC network have pivotal roles in the immune mechanism underlying sepsis. Subsequently, we identified module and hub genes (CD4 and STAT4) via construction of a sepsis immune-related PPI network. Then, we identified hub genes based on the modular structure of PPI network and generated a ceRNA subnetwork to analyze key lncRNAs associated with sepsis. Finally, 6 lncRNAs (LINC00265, LINC00893, NDUFA6-AS1, NOP14-AS1, PRKCQ-AS1 and ZNF674-AS1) that identified as immune biomarkers of sepsis. Moreover, the CIBERSORT algorithm and the infiltration of circulating immune cells types were performed to identify the inflammatory state of sepsis. Correlation analyses between immune cells and sepsis immune biomarkers showed that the LINC00265 was strongly positive correlated with the macrophages M2 (r = 0.77).

**Conclusion:**

Collectively, these results may suggest that these lncRNAs (LINC00265, LINC00893, NDUFA6-AS1, NOP14-AS1, PRKCQ-AS1 and ZNF674-AS1) played important roles in the immune pathogenesis of sepsis and provide potential therapeutic targets for further researches on immune therapy treatment in patients with sepsis.

**Supplementary Information:**

The online version contains supplementary material available at 10.1186/s12864-023-09535-7.

## Background

Sepsis is a systemic immune-inflammatory response caused by dysfunction of the body’s response to infection, which can lead to the multiple organ dysfunction syndrome (MODS) that seriously threatens the life safety of patients [1, 2]. In the treatment of sepsis, several novel approaches have been applied to ameliorate disease progression, including fluid resuscitation, symptom control, anti-infection and prevention of complications [3], as well as continuous blood purification [4] and extracorporeal membrane oxygenation (ECMO) [5]. The pathogenesis and progression of sepsis is extremely complex, which include imbalance of immune homeostasis, excessive inflammation response, cell death and abnormal molecular regulation all participate in the pathogenesis of sepsis [6]. Early diagnosis of sepsis is critical for timely treatment and improved sepsis outcomes [7]. However, at present, there are no effective measures for early diagnosis and intervention of sepsis [8]. Therefore, finding biomarkers for early intervention and diagnosis of sepsis may be important to reduce the mortality of sepsis patients as well as provide etiologic insight into sepsis.

Long non-coding RNAs (lncRNAs) range in size from 200 nucleotides to thousands of nucleotides. LncRNAs lack the ability to serve as protein templates, but they regulate the expression of genes and have variety of important biological regulatory functions [9]. Moreover, competing endogenous RNA (ceRNA) is a newly discovered regulatory mechanism by which RNA molecules (such as mRNA and lncRNA) targeted by common miRNAs can indirectly regulate each other through competition with miRNA response elements [10]. Emerging evidences indicate that lncRNAs may act as ceRNAs regulating risk genes and play a regulatory role in the development of various diseases. Ye et al. revealed that lncRNA NALT1 could acting as a ceRNA by binding with miR-574-5p to regulate the expression of the PEG10, thereby promoting colorectal cancer proliferation and migration [11]. In addition, Wang et al. found that the lncRNA KCNQ1OT1 played a crucial role in the inflammatory response and progression of acute lung injury due to sepsis by acting as a ceRNA to binding with miR-212-3p, thereby regulating MAPK1 expression and activating the p38/NF-κB pathway [12]. These results suggested that lncRNAs are involved in the sepsis-related immunoregulatory processes.

The immune-inflammatory response is the body’s defense against external injury. The immune-inflammatory response is a crucial pathological process in the patients with sepsis [13]. Recent studies have shown that ceRNA also regulate inflammatory response of the immune system in various diseases. For instance, Yan et al. identified that lncRNA HIX003209 promoted inflammation response by binding with miR-6089 via the TLR4/NF-κB pathway in patients with rheumatoid arthritis [14]. Moreover, lncRNAs-mediated ceRNAs also participate in immune-inflammatory response in infectious diseases. Wang et al. found lncRNA SHNG16 was shown to regulate the expression of TLR4 by inhibiting the miR-15a/16 cluster, which in turn affected lipopolysaccharide-induced inflammatory pathway in neonatal sepsis [15]. Even so, the roles of lncRNAs acting as ceRNAs in the mechanism of immunoregulatory processes underlying sepsis still remain unclear.

With the development of science and technology and the maturity of big data acquisition methods, correlation analysis based on bioinformatics technology enables us to have a certain understanding of the occurrence and development mechanism of patients with sepsis. In recent years, several studies have shown immune-related genes that involved in inflammatory processes could be latent therapeutic targets or diagnostic biomarkers in sepsis [16–18]. Dai et al. identified that LPIN1 was found to be a reliable biomarker for survival in patients with sepsis by using weighted gene co-expression network analysis (WGCNA) analysis and verified by qPCR [16]. Wang et al. identified 6 key genes (FYN, FBL, ATM, WDR75, FOXO1 and ITK) in sepsis through WGCNA analysis and PPI network analysis [18]. However, there is little known about the functions of lncRNAs which may thus be novel regulators of the pathogenesis of sepsis. To address this point, based on multi-step bioinformatics computational approach and ceRNA and PPI networks analysis, it is possible to predict more novel lncRNAs involved in immune responses after sepsis.

**Fig. 1:**
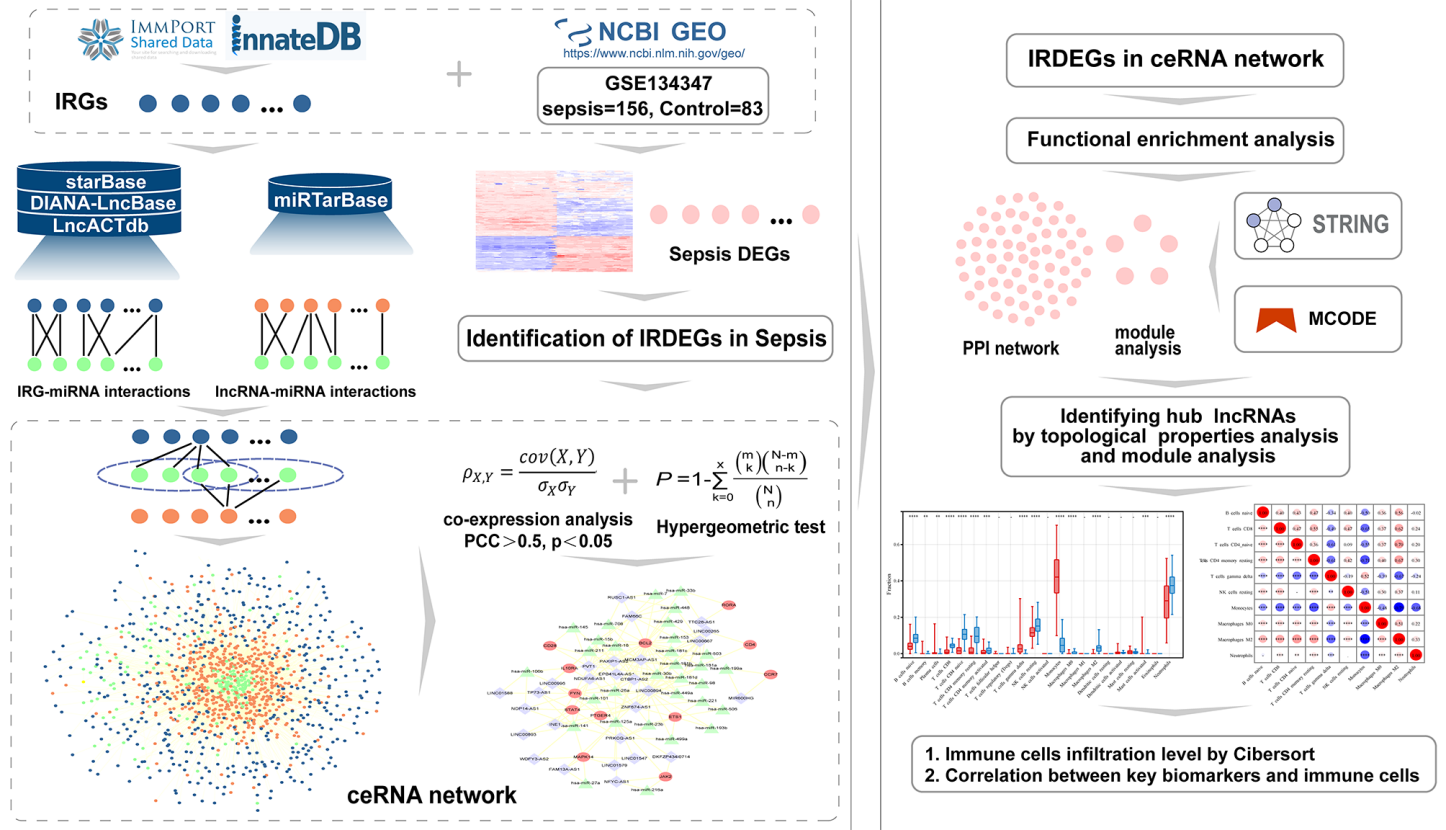
The flow chart of the research analysis process

In our present study, we constructed a global immune-associated ceRNA (IMCE) network based on data describing interactions pairs of gene–miRNA and miRNA–lncRNA. Next, we excavated a dysregulated sepsis immune-associated ceRNA (SPIMC) network from the global IMCE network by means of a multi-step computational approach, with a focus on gene expression profiles in sepsis. Enrichment assessments was applied to identify the roles of lncRNAs in SPIMC network were mainly related with inflammation and immune response. Subsequently, we used bioinformatics to generate and analyze PPI network and modular structure, to identify module and hub genes related to inflammatory and immunological status of patients with sepsis. Moreover, to further verify reliability of our results, we applied two machine learning algorithms (LASSO [19] and SVM-RFE [20]) to further filter and verification of the hub genes. Then, we obtained six hub lncRNAs that regulated the key genes based on ceRNA theory, which were identified as important regulatory components and potential key biomarkers for sepsis. Finally, we established the characteristics between these feature biomarkers and 22 kinds of infiltrating immune cells in each sample by using the CIBERSORT algorithm (flowchart shown in Fig. 1). Our study will provide new direction into the pathogenesis of sepsis and enhance the understanding of the role of ceRNA-mediated regulation of inflammation and immune responses in sepsis with the ultimate aim of providing a rationale for effective therapeutic targets in sepsis.

## Materials and methods

### Identification of immune-related genes

Immune-related genes (IRGs) were collected from the ImmPort**–**Immunology Database and Analysis Portal [21] and InnateDB [22] databases, which contain information concerning genes involved in the human innate immune response. Thus, we obtained 1793 IRGs from the ImmPort and 1040 IRGs from the InnateDB database. In total, we collected 2519 IRGs from the two databases.

### Gene–miRNA and miRNA–lncRNA interaction data

miRNA–mRNA interaction pairs data were obtained from miRTarBase (Release 7.0) [23], which contains information concerning experimentally verified gene–miRNA interactions that has been compiled from published experiments. We downloaded high-confidence functional (e.g. Luciferase reporter assay, Immunoblot, Western blot, AGO2 binding RNA immunoprecipitation qRT-PCR) miRNA–mRNA interaction pairs; this yielded 2843 genes and 740 miRNAs. Subsequently, we identified miRNA–lncRNA interaction pairs from starBase database [24], DIANA-LncBase database [25], and LncACTdb databases [26], which contain high-throughput and experimentally validated interaction pairs (e.g. HITS-CLIP, iCLIP, CHIP, PAR-CLIP, CLASH). In total, we obtained 644 miRNAs and 582 lncRNAs from combined analysis of the above miRNA–lncRNA interaction data. Then, based on miRBase [27] and RNAcentral [28] databases, we unified miRNAs naming to subsequent processing.

### mRNA expression profiles for sepsis and data processing

The GSE134347 datasets were downloaded from the Gene Expression Omnibus database. GSE134347 featured data acquired from the peripheral whole blood of 156 sepsis patients and 83 healthy controls (platform: GPL17586). The R package “stats” was used to principal component analysis (PCA) by using the “prcomp” function. The “limma” package was used to identify differentially expressed genes (DEGs) between sepsis patients and healthy control samples, with the screening criteria |log2 fold change|>1.0 and adjusted P-value < 0.05. The intersection of IRGs with DEGs yielded IRDEGs. We used the “ComplexHeatmap” R package to generate heatmap and the “gplots” R package to generate volcano plots. Furthermore, the lncRNA profile under accession number GSE217700 was from 4 sepsis patients and 4 healthy controls and was used for validation dataset.

### Construction of a global IMCE network

We constructed a global IMCE network based on ceRNA theory that competing mRNA–lncRNA interaction pairs share common miRNA binding sites [28]. For a given immune-related ceRNA interaction pair, lncRNAs and IRGs share common miRNAs, forming a competing triad. Then, we constructed a global IMCE network after all lncRNA–miRNA–IRG interaction pairs had been assembled and visualized in Cytoscape software. In the network, the nodes represented IRGs, miRNAs, and lncRNAs, while the edges represented their interactions. Moreover, we analyzed the topological features for all nodes in the global IMCE network.

### Hypergeometric test

We established the dysregulated SPIMC network from the global IMCE network by hypergeometric test and co-expression correlation analysis. The hypergeometric test was used to evaluate the significance of the shared miRNAs between each mRNA and lncRNA, mRNA-lncRNA interaction pairs with a P-value < 0.01 were considered to be statistically significant. We applied the cumulative hypergeometric test and computed P-values using the following formula:

$$P=1-\sum _{k=0}^{x}\frac{\left(\genfrac{}{}{0pt}{}{m}{k}\right)\left(\genfrac{}{}{0pt}{}{N-m}{n-k}\right)}{\left(\genfrac{}{}{0pt}{}{N}{n}\right)}$$

For each interaction pair, the total number of miRNAs in the interaction data were presented as *N*, the number of miRNAs that were associated with one lncRNA and one mRNA were presented as n and m, and the number of miRNAs shared with the lncRNA and mRNA were presented as x.

### Pearson correlation coefficients analysis (PCC)

Next, we applied co-expression analysis for mRNA–lncRNA interaction pairs by using PCC to examine the expression patterns of IRDEGs and lncRNAs. PCC was used to measure the correlation between the expression levels of two variables. The expression data of the lncRNAs and genes were downloaded from the Genotype-Tissue Expression (GTEx, v8 release) [29] and PCC were calculated by using the following formula:

$${\rho }_{X,Y}=\frac{cov\left(X,Y\right)}{{\sigma }_{X}{\sigma }_{Y}}$$

Here, σ_*X*_ and σ_*Y*_ represent standard deviations for *X* and *Y*, while cov *(X, Y)* refers to the covariance of variables *X* and *Y*. Finally, the co-expressed interaction pairs that met the PCC threshold (PCC > 0.7 and P < 0.01) and crossed the hypergeometric test threshold (P < 0.01) were regarded as statistically significant interaction pairs. After integrating all of the competing triplets, we constructed the SPIMC network. We constructed and visualized SPIMC network by using Cytoscape v3.8.1.

### Construction of immune-related PPI network and module analysis

It is well known that genes play an important role in the pathogenesis of sepsis. LncRNAs lack the ability to serve as protein templates, but they regulate the expression of genes and have variety of important biological regulatory functions. To identify the interactions of proteins encoded by sepsis-related genes, we constructed a PPI network of IRDEGs in the SPIMC. Furthermore, module analysis was performed to find the key genes shared common properties and related to inflammatory and immunological status of patients with sepsis. Immune-related PPI network analysis was then performed to interpret the molecular mechanisms of key cellular activities. In this study, PPI of IRDEGs with interaction scores ≥ 0.4 in the SPIMC network were determined by using Search Tool for the Retrieval of Interacting Genes database (STRING v11.0) [30] and visualized with Cytoscape. Furthermore, the Molecular Complex Detection (MCODE) tool [31] in Cytoscape software was utilized to identify highly interconnected modules and functionally related from the PPI network using selection criteria (MCODE degree cutoff = 2; k-core = 2; max. depth = 100; node score cutoff = 0.2).

### Functional enrichment assessments

To determine the potential functions of the lncRNAs in SPIMC network, we performed functional enrichment analyses of KEGG pathways [32–34] and GO functions based on co-expressed IRDEGs by using “clusterProfiler” R package [35]. The KEGG pathway and GO database generated from “org.Hs.eg.db” R package. The adjusted P-values < 0.01 were considered as significantly GO terms and KEGG pathways, and then these results were visualized by “Ggplot” R package.

### Identifying of key immune-related lncRNAs of sepsis and evaluation of immune cell subtypes distribution

Then we characterized the topological properties of PPI network and identified one module that contained 6 IRDEGs and in which CD4 and STAT4 had higher degrees in PPI network. The hub IRDEGs in sepsis which were obtained by PPI network and module analysis were subsequently overlapped within the SPIMC network. To evaluate the hub lncRNAs regulation as ceRNAs in sepsis, a lncRNA-mediated module-associated ceRNA network was constructed. The ceRNA subnetwork that included hub lncRNAs was then defined as potential sepsis immune-relevant lncRNA. Next, we performed infiltrating fractions of immune cells based on the GSE134347 datasets of sepsis by CIBERSORT algorithm [36] (https://cibersortx.stanford.edu), which with 22 kinds of immune cell with using 1,000 permutations to estimate the relative abundance of immune cells infiltration. We filtered the samples for P-values < 0.05 in the CIBERSORT as statistically significant results. Then, we used Spearman’s correlation analysis to estimate the correlation between the degrees of immune cells infiltration with significant differences between groups. Finally, these results were analyzed and visualized by the “Corrplot” and “Ggplot2” R package.

### Evaluation the correlation between biomarkers and immune cells infiltration

Finally, we estimated the characteristics between these feature lncRNAs biomarkers and 22 kinds of infiltrating immune cells. The correlation between the immune-related lncRNAs co-expression IRDEGs expression and levels of infiltrating fractions of immune cells were evaluated by the R language based on Spearman’s correlation and visualized with “ggplot2” R package.

## Results

### The global IMCE network construction and topological analysis

**Fig. 2:**
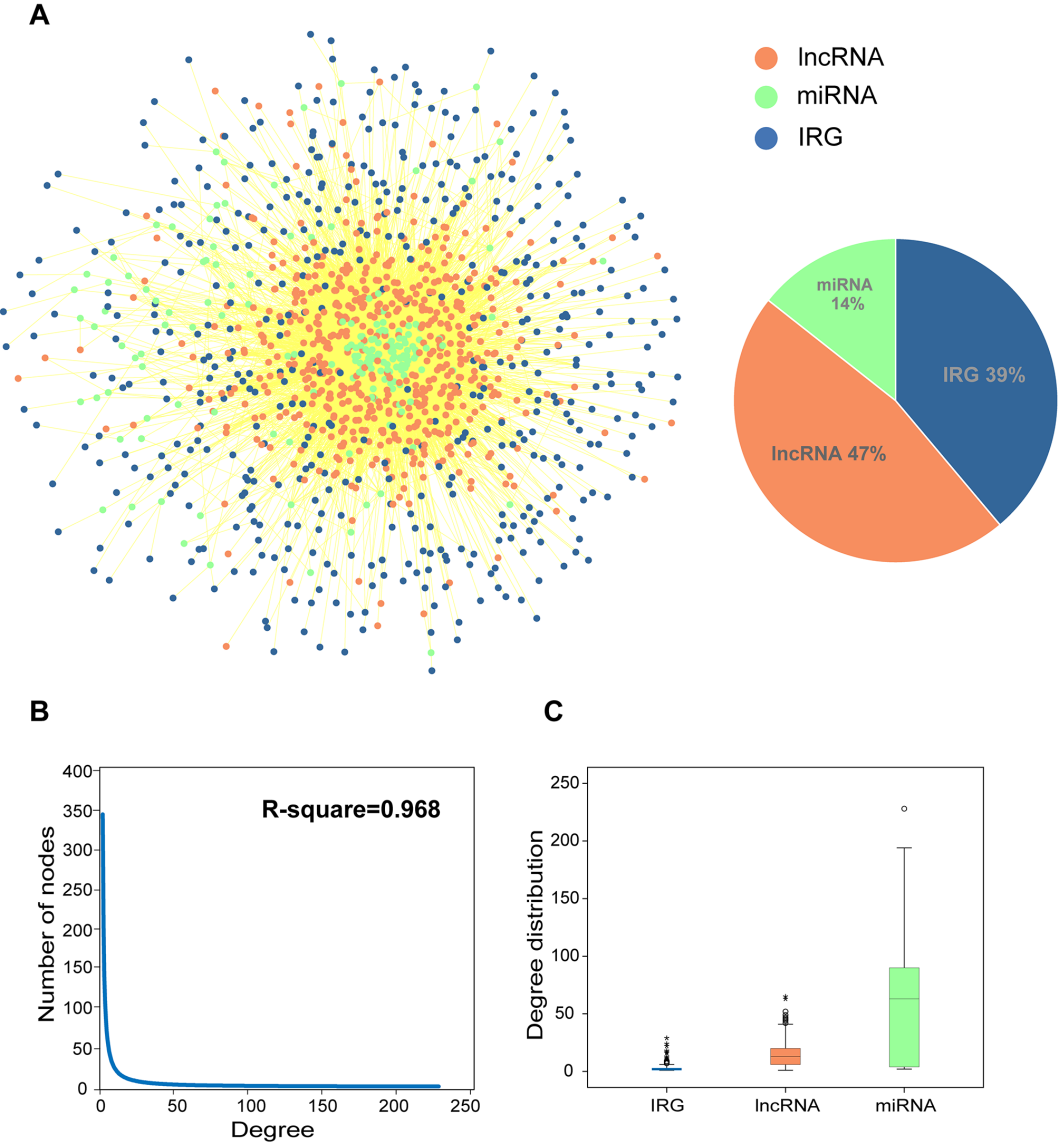
Construction and analysis of the global IMCE network. (**A**) The global IMCE network. Blue, green, and orange nodes represent IRGs, miRNAs, and lncRNAs, respectively. Lines between IRGs, miRNAs, and lncRNAs represent their interactions. The pie chart shows the number of IRGs, miRNAs, and lncRNAs in the network. (**B**) The nodes degree distribution of the global IMCE network. (**C**) The degree distribution of IRGs, miRNAs and lncRNAs in global IMCE network

To construct a global IMCE network, we obtained human IRGs from the ImmPort database and InnateDB database, along with human miRNA–gene and miRNA–lncRNA interaction pairs from the miRTarBase database, starBase database, DIANA-LncBase database, and LncACTdb database. We constructed a network by integrating the abovementioned data (Fig. 2A). The global IMCE network contained 1232 nodes (479 IRGs, 576 lncRNAs, and 177 miRNAs) and 9655 edges. We found that many nodes were lncRNAs, suggesting essential roles of lncRNAs in the network. Subsequently, we analyzed the nodes degree distribution in the network; nodes in the global IMCE network closely followed the power law distribution (f(x) = 346.86x-1.21, R^2^ = 0.968), which suggested that network is approximately scale-free network (Fig. 2B). Moreover, comparative analysis revealed significant differences in degree distribution among the miRNAs, mRNAs, and lncRNAs (P < 0.05) (Fig. 2C). These findings indicated that both lncRNAs and miRNAs exhibit considerably high degrees, suggesting that they have important roles in the network. Collectively, the global IMCE network can serve as a starting point for investigating of the immune processes involved in sepsis.

### Identification of DEGs and sepsis-associated IRDEGs

**Fig. 3:**
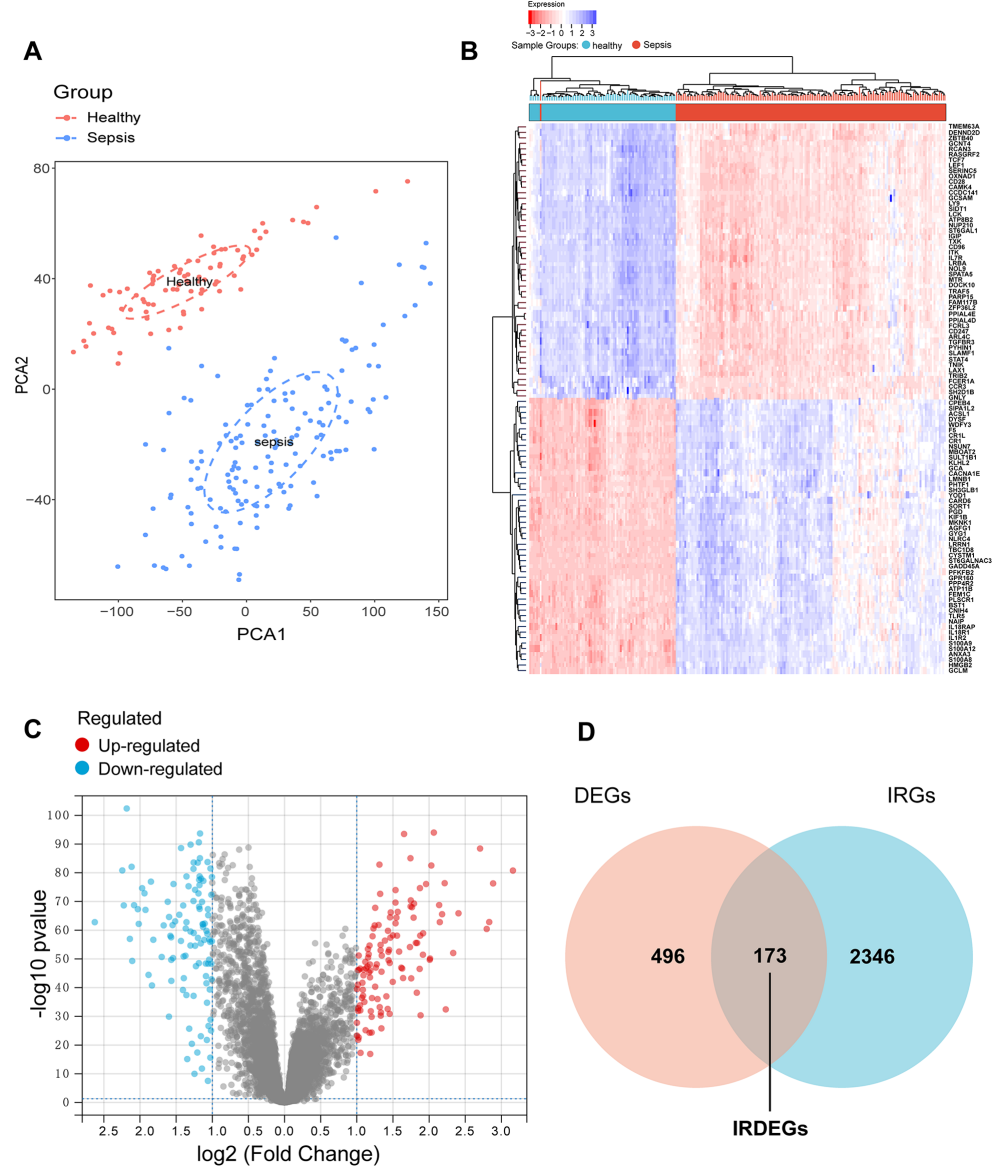
Identification of DEGs and immune-related DEGs in sepsis. (**A**) The principal component analysis results of the GSE134347 expression matrices. (**B**) Hierarchical heatmap for DEGs in sepsis patients and healthy controls. (**C**) Volcano plot for DEGs in sepsis patients and healthy controls (**D**) Venn diagram of the intersection of the DEGs and IRGs; the intersection represents overlapping genes (IRDEGs).

The expression files between sepsis groups and control groups in the GSE134347 were normalized and identified using “limma” R package with |log2 FC|>1.0 and adjusted P-value < 0.05. The PCA plot of the GSE134347 dataset was shown in Fig. 3A. As a result, we successfully identified 317 genes were up-regulated and 352 genes were down-regulated in GSE134347 dataset. The heatmap and volcano plots were shown to depict the expression tendencies and distribution of these DEGs between sepsis and controls (Fig. 3B, C). Because the immune response has important roles in the pathogenesis of sepsis [37], we then focused on the sepsis-associated IRDEGs that were identified by the intersection of the IRGs and DEGs. Finally, we obtained 173 sepsis-associated IRDEGs in the two gene sets mentioned above (Fig. 3D).

### Excavation of a SPIMC network from the global IMCE network

**Fig. 4:**
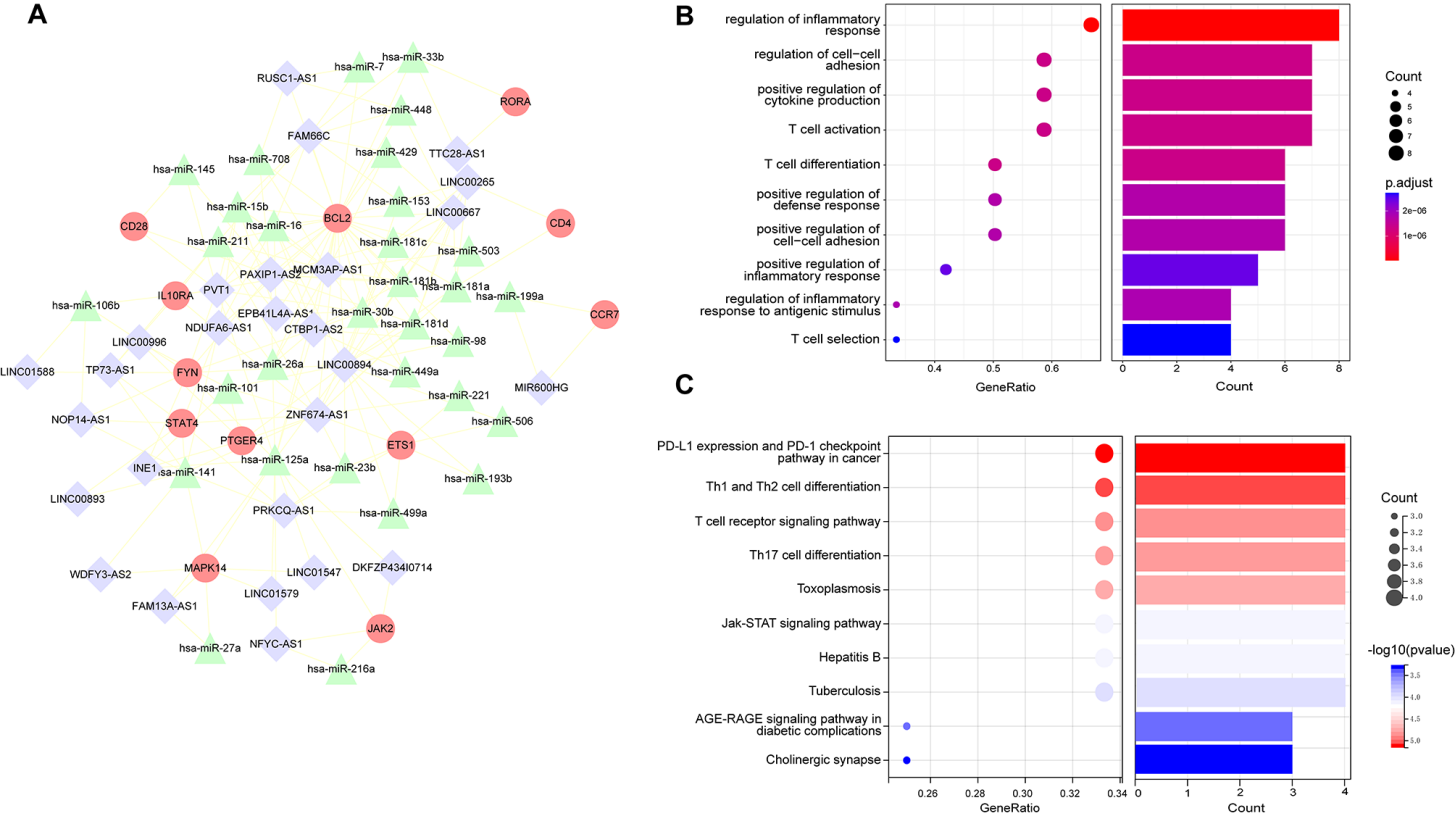
Excavated and analysis of the SPIMC network. (**A**) Construction of the SPIMC network in sepsis. Red circles represent IRDEGs, green triangles represent miRNAs, and purple diamond represent lncRNAs. Lines represent interactions among them. (**B**) The GO function analysis of IRDEGs regulated by lncRNAs in the SPIMC network. The degree of enrichment increases from blue to red. The bigger circles suggest a more significant proportion of genes among GO function genes. (**C**) Pathway enrichment analysis of IRDEGs regulated by lncRNAs in the SPIMC network. The degree of enrichment increases significantly from blue to red. The bigger circles suggest a more significant proportion of genes among KEGG pathway genes

To identify the relationships between IRDEGs, miRNAs and lncRNAs, as well as exploring key lncRNAs in sepsis, we mapped 173 IRDEGs to the global IMCE network. As a result, we mapped 100 gene–miRNA interaction pairs and 4254 lncRNA-miRNA interaction pairs. Next, we performed hypergeometric tests and PCC analysis, setting the thresholds with PCC > 0.7 and P-value < 0.01 to meet statistical significance of the IRDEGs-miRNAs-lncRNAs interactions. Then, a significantly dysregulated SPIMC network (Fig. 4A) was established which contained 70 nodes (12 IRDEGs, 27 lncRNAs and 31 miRNAs) and 189 edges (as listed in Table 1).

**Table 1:** The information of SPIMC network

IRDEGs	BCL2, CCR7, CD28, CD4, ETS1, FYN, IL10RA, JAK2, MAPK14, PTGER4, RORA, STAT4
miRNAs	miR-181b, miR-27a, miR-141, miR-101, miR-26a, miR-15b, miR-181d, miR-16, miR-125a, miR-98, miR-30b, miR-449a, miR-211, miR-153, miR-429, miR-7, miR-33b, miR-448, miR-181a, miR-503, miR-708, miR-199a, miR-145, miR-499a, miR-23b, miR-506, miR-193b, miR-221, miR-106b, miR-216a, miR-181c
LncRNAs	CTBP1-AS2, EPB41L4A-AS1, FAM66C, LINC00265, LINC00667, LINC00894, MCM3AP-AS1, NDUFA6-AS1, PAXIP1-AS2, PVT1, RUSC1-AS1, ZNF674-AS1, MIR600HG, PRKCQ-AS1, INE1, LINC01588, NOP14-AS1, TP73-AS1, DKFZP434I0714, NFYC-AS1, FAM13A-AS1, LINC01547, LINC01579, WDFY3-AS2, LINC00996, TTC28-AS1, LINC00893

### Enrichment analysis of IRDEGs in SPIMC network

To explore the biological functions of lncRNAs in the SPIMC network, we conducted GO enrichment analysis and KEGG pathway through the lncRNAs co-expressed IRDEGs. In total, we obtained 553 GO terms (detail information in supplementary Table S1) and 41 KEGG enriched pathways (detail information in supplementary Table S2). The top 10 significant KEGG pathways and GO functional enrichment terms (P-value < 0.05) which might play pivotal roles in the immunological mechanisms of sepsis were shown in Fig. 4B, C. GO function analysis showed that these IRDEGs mostly enriched in biological processes related with regulation of inflammatory response (GO:0050727), T cell activation and differentiation (GO:0042110, GO:0030217), regulation of inflammatory response to antigenic stimulus (GO:0002861). KEGG pathway showed that they were mostly enriched in Jak-STAT signaling pathway (hsa04630), PD-L1 expression and PD-1 checkpoint pathway in cancer (hsa05235), T cell receptor signaling pathway (hsa04660). Our findings indicate that these IRDEGs co-expressed lncRNAs regulate multiple risk signaling pathways and may have various function roles in sepsis.

### Generation of sepsis immune-related PPI network and screening hub lncRNAs

**Fig. 5:**
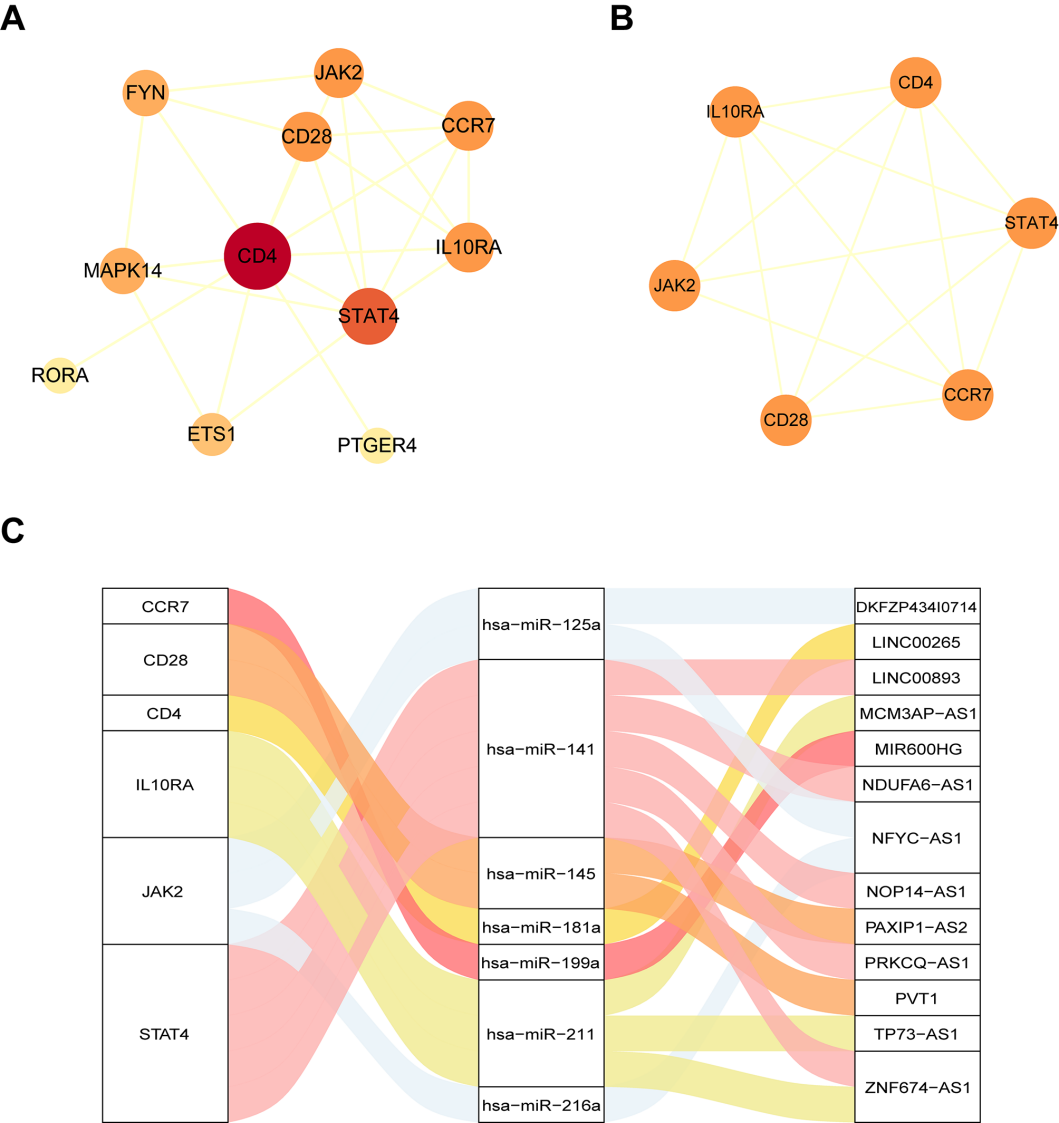
Construction of a PPI network and screening key candidate lncRNA for sepsis. (**A**) The sepsis immune-related PPI network. Orange circles represent IRDEGs. The bigger and darker the node is, the higher degree the node has. Lines represent their regulatory interactions. (**B**) The module from sepsis immune-related PPI network is modularized by MCODE. (**C**) The interactions between the lncRNAs, hub genes and miRNAs. The first column represents genes, the second column represents miRNAs and the third column represents lncRNAs.

Based on the SPIMC network we obtained 12 IRDEGs, and then the STRING database was used to detect potential interactions of the IRDEGs. Accordingly, a total of 25 PPI interactions were identified and used to construct immune-related PPI network (Fig. 5A). Then, we used MCODE to analyze the network and obtained one module which contained 6 IRDEGs (CD4, STAT4, CD28, IL10RA, CCR7 and JAK2) (Fig. 5B). Moreover, based on the ceRNA hypothesis, we combined the hub IRDEGs and the lncRNA–miRNA–mRNA interactions between them (Fig. 5C); the resulting module contained 6 IRDEGs, 7 miRNAs and 13 lncRNAs. Furthermore, CD4 and STAT4 had higher degrees in the PPI network and have been reported participate in immune mechanisms underlying sepsis, which indicated their important regulate roles in sepsis immune processes. Therefore, CD4 and STAT4 were considered to be hub genes of sepsis, and 6 lncRNAs (LINC00265, LINC00893, NDUFA6-AS1, NOP14-AS1, PRKCQ-AS1 and ZNF674-AS1) that regulated the two genes abovementioned based on ceRNA theory were identified as potential key biomarkers of sepsis. To further verify the reliability of our results, we downloaded another lncRNA expression profile (GSE217700) of sepsis from GEO database. By analyzing the high-throughput lncRNA expression profile, 883 DElncRNAs were identified (Supplementary Table S3). There were two common shared DElncRNAs, namely LINC00265 and PRKCQ-AS1. The common shared DElncRNAs between 6 candidate lncRNAs and GSE217700 were statistically significant (*P* < 0.05) based on a hypergeometric test (Supplementary Figure S1). These findings further enhanced the credibility of our results. Collectively, these findings indicate that these hub lncRNAs were involved in the immunological and inflammatory processes underlying the pathogenesis of sepsis.

### Evaluation of the circulating immune cell infiltration

**Fig. 6:**
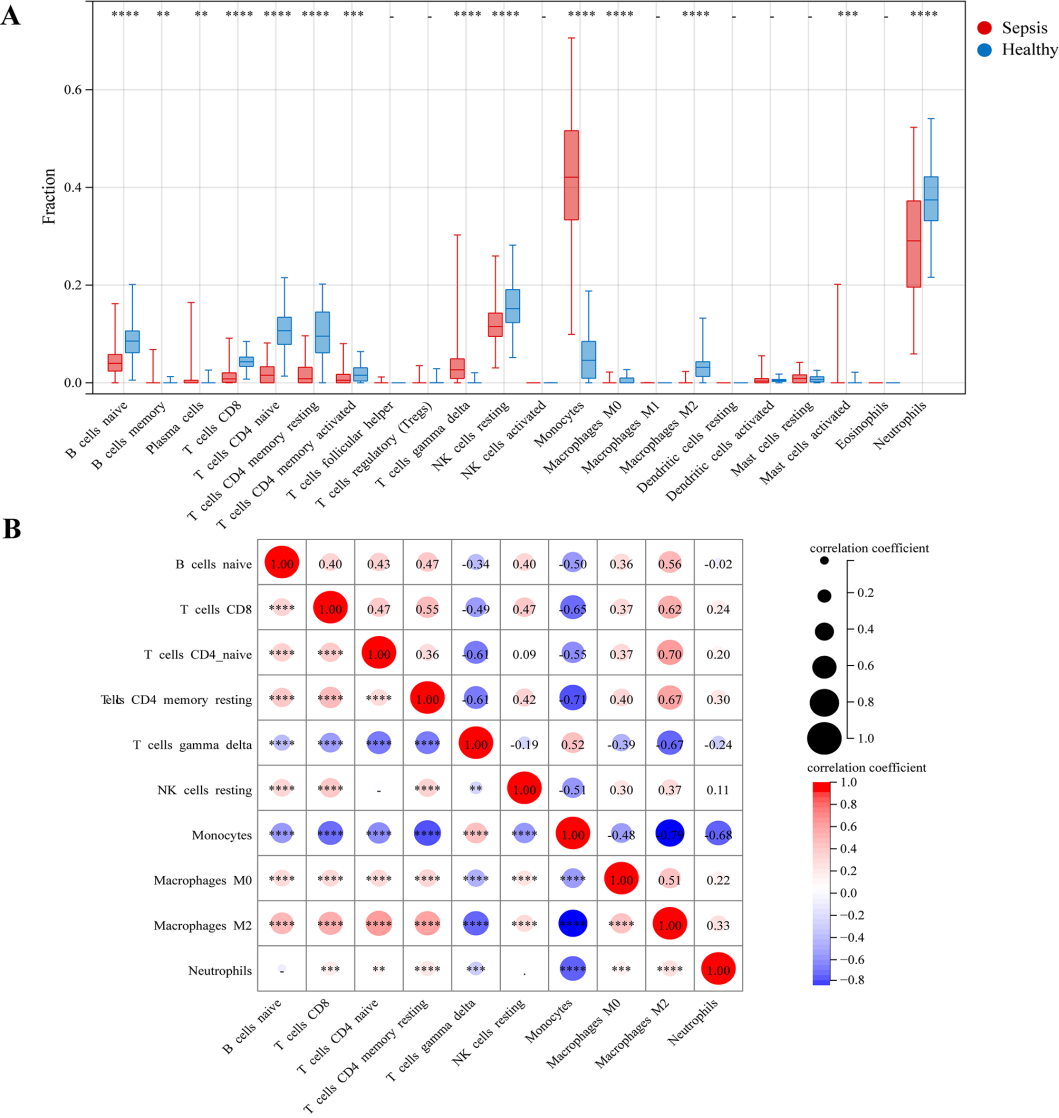
Immune cell infiltration analysis in sepsis. (**A**) Box plot of 22 kinds of immune cells infiltration between sepsis patients and healthy controls. Blue and red colors represent normal and sepsis samples, respectively. (**B**) The correlation analysis of the immune cells with significant differences. Red suggests the positive correlation, blue suggests the negative correlation, and a darker colour suggests a stronger correlation. ^*^P < 0.05, ^**^P < 0.01, ^***^P < 0.001, ^****^P < 0.0001

The infiltration of 22 immune cells types was calculated in different samples of sepsis and controls by CIBERSORT immune subset deconvolution. The box plot presents the 22 immune cells types infiltration both in samples of sepsis and healthy controls (Fig. 6A). The sepsis groups generally stored a higher proportion of B cells memory (*P* = 0.0015), plasma cells (*P* = 0.0011), T cells gamma delta (*P* < 0.0001), monocytes (*P* < 0.0001), mast cell activated (*P* < 0.001), while the proportions of B cells naive (*P* < 0.0001), CD8^+^ T cells (*P* < 0.001), naive CD4^+^ T cells (*P* < 0.0001), memory resting CD4^+^ T cells (*P* < 0.0001), memory activated CD4^+^ T cells (*P* < 0.001), NK cells resting (*P* < 0.0001), macrophages M0, macrophages M2 (*P* < 0.0001) and neutrophils (*P* < 0.0001) were relatively lower. Then, we estimated the correlation between significant differences immune cell types in the degree of infiltration between sepsis and healthy. As the correlation heatmap shown in (Fig. 6B), monocytes and macrophages M2 (Spearman’s Corr = − 0.79), memory resting CD4^+^ T cells and monocytes (Spearman’s Corr = − 0.71), monocytes and neutrophils (Spearman’s Corr = − 0.68, p < 0.0001) showed the significant negative correlations, respectively. M2 macrophages and naive CD4^+^ T cells (Spearman’s Corr = 0.70), memory resting CD4^+^ T cells and M2 macrophages (Spearman’s Corr = 0.67), CD8^+^ T cells and M2 macrophages (Spearman’s Corr = 0.62) showed the significant positive correlations, respectively.

### Analysis of correlation between the feature lncRNAs and immune cells

**Fig. 7:**
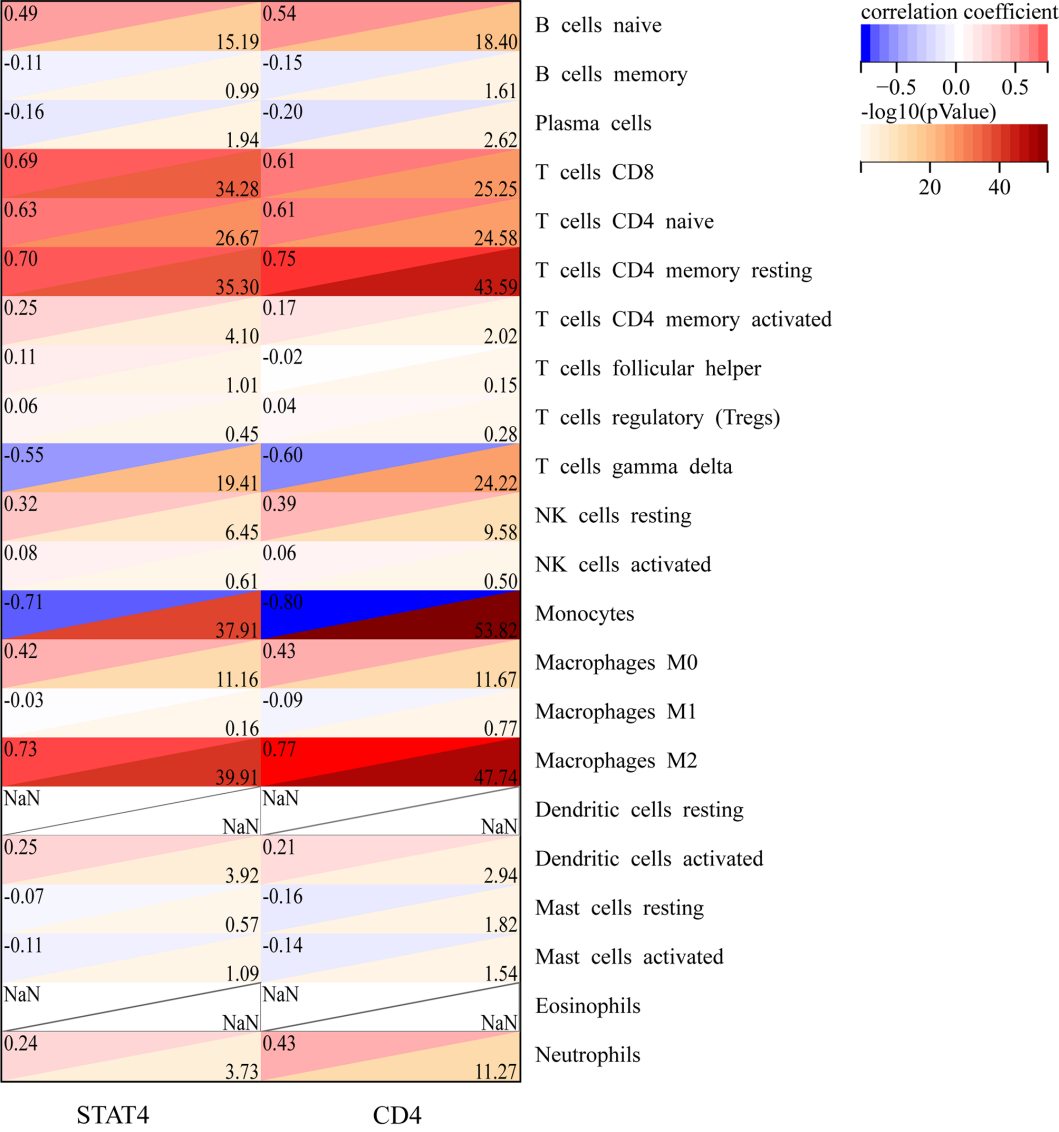
Heatmap showing the correlation between feature lncRNAs and immune cells. The correlation coefficients were estimated by using Spearman’s correlation analysis; Red suggests the positive correlation, blue suggests the negative correlation, and a darker colour suggests a stronger correlation

Finally, we estimated the correlation between the feature lncRNAs and 22 types of immune cells based on the co-expressed IRDEGs. The correlation was evaluated by using Spearman’s correlation coefficient based on the R software. The expression of CD4 displayed a strong positive correlation with Macrophages M2 and memory resting CD4^+^ T cells and displayed a strong negative correlation with Monocytes and T cells gamma delta. While, the expression of STAT4 displayed a strong positive correlation with Macrophages M2, memory resting CD4^+^ T cells and CD8^+^ T cells and displayed a strong negative correlation with T cells gamma delta and monocytes (Fig. 7). Furthermore, what is noteworthy is that CD4 was strongly positive correlated with the proportion of macrophages M2 (r = 0.77).

## Discussion

At present, the pathogenesis of sepsis is yet to be elucidated, and there is a lack of timely diagnosis methods for sepsis. Recent researches have demonstrated that sepsis involves in the activation of immune responses [38] which is associated with the up-regulated of chemokines [39], proinflammatory cytokines [39], proteins released from activated platelets and neutrophils, coagulation factors and complement products [40, 41]. Immune response is the main pathological process of sepsis and participates in the regulation of sepsis process [42, 43]. Therefore, constructing the immune-associated ceRNA regulatory network could provide novel perspective on the cellular and molecular mechanisms of sepsis and help to elucidate the immune mechanism of sepsis. In present study, the integrated analysis and computational approach were performed by using gene expression profiles and experimentally verified interactions to explore immune-associated ceRNAs in sepsis.

We first investigated a global IMCE network based on the genes and lncRNAs compete for common miRNA. The highest degrees of lncRNAs (e.g. HAND2-AS1 and CASC2) in the network were identified as to be participated in immune or inflammatory responses in multiple diseases. Yang et al. found that HAND2-AS1 level was elevated in patients with hepatocellular carcinoma and its dysregulation related to immune response and metabolic processes [44]. LncRNA CASC2 was found to bind with miR-27b, and alleviated LPS‑induced injury in acute lung injury cell model by inhibiting apoptosis and inflammatory cytokine production [45]. Consequently, construction of the global network has shown that lncRNAs commonly modulate immune processes in various diseases; this provides an important background for the immunopathogenesis of sepsis.

We constructed a SPIMC network by mapping the IRDEGs to the global IMCE network and then identified the functions of lncRNAs. Functional enrichment analysis revealed that they can play important roles in sepsis development. The GO function results indicated that lncRNAs in SPIMC network were mostly involved in the biological processes related with regulation of inflammatory response (GO:0050727), T cell activation and differentiation (GO:0042110, GO:0030217), regulation of inflammatory response to antigenic stimulus (GO:0002861). Similarly, the results of KEGG pathways assessments showed that these lncRNAs co-expressed IRDEGs several enriched immune-related pathways. The results of the original dataset showed that immune inflammatory responses might play important roles in the development of sepsis [46]. Our findings also indicated that several significant inflammation or immune related biological pathways were closely concerned with sepsis, which is partially similar to the results of the original dataset. Our present findings suggested that the lncRNA might result in the abnormal expression of genes which might lead to the dysregulation of key pathways, thus leading to the occurrence and progression of sepsis. Therefore, these lncRNAs may play important roles in the immune mechanism of sepsis.

Modularization can dissect complex network into specific modules and the genes within these modules may play an important functional role. Then, we established a PPI network based on 12 IRDEGs that obtained from the SPIMC network and identified module and hub genes (detail information are shown in Table 2). Furthermore, by analyzing the network, the CD4 and STAT4 had higher degrees in the PPI network and we identified 6 hub lncRNAs-mediated ceRNA interaction pairs featuring the two hub genes. To further verify reliability of our results, based on the IRDEGs in the SPIMC network, we applied two machine learning algorithms (LASSO and SVM-RFE) to further filter the hub genes. As a result, 8 IRDEGs were obtained by the LASSO regression algorithm (Supplementary Figure S2A). Meanwhile, 8 IRDEGs were featured by SVM-RFE algorithm (Supplementary Figure S2B). There were 3 common shared IRDEGs (CD4, STAT4, CD28) between LASSO, SVM-REF and module analysis of PPI network (Supplementary Figure S2C). These findings enhanced the credibility of our results.

**Table 2:** Top 6 hub genes identified by MOCODE

Gene symbol	Node degree	Description
CD4	10	CD4 molecule
STAT4	7	signal transducer and activator of transcription 4
JAK2	5	Janus kinase 2
IL10RA	5	Interleukin 10 receptor subunit alpha
CD28	5	CD28 molecule
CCR7	5	C-C motif chemokine receptor 7

These key genes are not only hub genes but are IRDEGs in sepsis, that may be potential targets for sepsis regulation. Recently, several studies indicated that CD4 and STAT4 could be participated in the development of sepsis. The dysregulation expression of STAT4 genes in monocytes of sepsis patients may inhibit its therapeutic potential [47]. As a result, we obtained 6 lncRNAs as biomarkers (LINC00265, LINC00893, NDUFA6-AS1, NOP14-AS1, PRKCQ-AS1 and ZNF674-AS1) that were closely associated with immune and inflammatory response in sepsis. Previous research has shown that the expression of lncRNA LINC00265 was up-regulated in osteoarthritis patients and directly suppressed the expression of miR-101-3p; conversely, LINC00265 knockdown alleviated cell apoptosis and inflammation induced by caspase-3 in osteoarthritis [48]. Additionally, lncRNA LINC00893 regulated the suppressor of SOCS3/JAK2/STAT3 pathway by acting as a ceRNA to bind with miR-3173-5p in patient with prostate cancer [49]. The activation of JAK/STAT signaling pathways has diverse functions in the pathogenesis of sepsis [50]. Moreover, lncRNA ZNF674-AS1 was found that directly interacted with miR-181a to regulate the expression of SOCS4 [51]. SOCS4 gene may be a potential regulator to inhibit cytokine storm and cytokine overproduction in mouse models infected with the virus [52]. Though these lncRNAs have been demonstrated by previous researches are not specific for sepsis, these results elucidate that our methods for identifying immune-related lncRNAs in sepsis are reliable. The disturbance of immune homeostasis affects the occurrence and development of sepsis, so the early diagnosis of sepsis is particularly important. These findings will be helpful to identify new biomarkers for the diagnosis in patients with sepsis. However, the exact function of the other lncRNAs in the SPIMC network remains unknown, which may be the novel regulators in the immune pathogenesis of sepsis and requires further research to be fully clarified.

Increasing evidence documented that immune cells undergo numerical and functional abnormalities in the pathogenesis of sepsis [53, 54]. Thus, we determined the immune subsets infiltration between healthy controls and sepsis patients by CIBERSORT algorithm. The results of circulating immune cell infiltration showed the higher proportions of B cells memory, plasma cells, T cells gamma delta, monocytes and mast cell activated in sepsis group, while with lower proportions of B cells naive, CD8^+^ T cells, naive CD4^+^ T cells, memory activated CD4^+^ T cells, memory resting CD4^+^ T cells, NK cells resting, macrophages M0, macrophages M2 and neutrophils, indicating these cells may be related to the progression and occurrence of sepsis. Interestingly, we found that T cell subsets were significantly correlated with sepsis, and the proportion of naive CD4^+^ T cells, memory activated CD4^+^ T cells, memory resting CD4^+^ T cells and CD8^+^ T cells in sepsis patients decreased significantly. These results consistent with the novel viewpoint about sepsis. Previous study efforts have highlighted that the immunosuppression and immune activation throughout the course of the sepsis [55, 56]. Dysfunction of the innate immune system and immunosuppression of the adaptive immune system could lead to the unbalanced and persistent inflammatory and anti-inflammatory responses [54–56]. Therefore, maintaining the immune system homeostasis and studying the molecular regulation mechanism of lncRNA in T cells in the pathogenesis of sepsis will help us develop new therapies targeting lymphocytes or cytokines to improve patient prognosis and reduce mortality.

Furthermore, we determined the correlations of feature lncRNAs co-expressed genes and infiltrating immune cells by Spearman’s correlation analysis. Strikingly, LINC00265 co-expressed gene CD4 was strongly correlated with the proportion of M2 macrophages. Of note, LINC00265/miR-101-3p/TOP2A have been found to regulate the immune checkpoints expression and modulate the immune status by positively correlated with macrophages in hepatocellular carcinoma [57]. However, there were still some limitations in our methods. Because of the inconsistent symbols of database, a great deal of mRNAs and lncRNAs might be lost during the process of integrating the data, which may decrease our result unfortunately. Therefore, we mapped these miRNAs to miRBase [27] and RNAcentral [28] for uniform naming, thus trying to make the data more accurate. Moreover, the information about these sophisticated lncRNAs and immune cells interacting processes are currently lacking, identifying the molecular mechanisms and functional of immune cell infiltration in sepsis would be future research direction. Finally, because of the small number of samples from patients with sepsis, some identified lncRNAs may be false positives. In future studies, we will continue to concentrate and collecting experimental data to further explore the in vivo roles of lncRNAs in sepsis pathogenesis.

## Conclusion

In conclusion, we identified 6 lncRNAs (LINC00265, LINC00893, NDUFA6-AS1, NOP14-AS1, PRKCQ-AS1 and ZNF674-AS1) related to the immune pathogenesis of sepsis and LINC00265 was strongly correlated with the proportion of M2 macrophages. The global IMCE and SPIMC network constructed in our present research could provide a comprehensive strategy for excavating the molecular basis of sepsis. Our study is the first to comprehensively analysis to the molecular biological characteristics in the immune process of sepsis and presented a new direction to identify immune-related biomarkers in sepsis, which may serve as the potential therapeutic targets for immune therapy treatment in patients with sepsis.

### Electronic supplementary material

Below is the link to the electronic supplementary material.

**Additional file 1: Supplementary Table S1**. GO functional enrichment assessments of IRDEGs regulated by lncRNAs in SPIMC network

**Additional file 2: Supplementary Table S2**. KEGG pathway enrichment assessments of IRDEGs regulated by lncRNAs in SPIMC network

**Additional file 3: Supplementary Table S3**. Differentially expressed lncRNAs in the GSE217700 dataset

**Additional file 4: Supplementary Figure S1**. Validation of the DElncRNAs in GSE217700 dataset. (A) The volcano plot of DElncRNAs in GSE217700 dataset. (B) Box plots of LINC00265 and PRKCQAS1 expression between sepsis patients and normal controls in GSE217700. (C) Venn diagram of 6 hub lncRNAs and GSE217700; red ellipse indicates 6 candidate lncRNAs, blue ellipse indicates DElncRNAs of GSE217700; overlap intersection indicates common shared DElncRNAs

**Additional file 5: Supplementary Figure S2**. Machine learning in the identification of hub genes. (A, B) Identified optimal IRDEGs by using LASSO regression and SVM algorithms. (C) The intersection of candidate hub genes between LASSO, SVM and PPI module analysis

Supplementary Material 6

## Data Availability

The data used to analyze in this study could be found in the GEO database: https://www.ncbi.nlm.nih.gov/geo/GSE134347. The original contributions presented in the study and the code are included in the article and supplementary material, further questions and reasonable request for the code can be directed to the corresponding author.
