# Supplementary material for: Identification of immune-related lncRNA in sepsis by construction of ceRNA network and integrating bioinformatic analysis
Source: BMC Genomics. 2023 Aug 24;24:484. doi: 10.1186/s12864-023-09535-7 (PMC10464037; doi:10.1186/s12864-023-09535-7)
Supplement: Supplementary file 1 — Additional file 1: Supplementary Table S1. GO functional enrichment assessments of IRDEGs regulated by lncRNAs in SPIMC network [file 12864_2023_9535_MOESM1_ESM.docx]

**Additional files:**

**Additional file 1:**

Supplementary Table 1: GO functional enrichment assessments of IRDEGs regulated by lncRNAs in SPIMC network.

| ONTOLOGY | ID | Description | p-value | p.adjust |
| --- | --- | --- | --- | --- |
| BP | GO:0050727 | regulation of inflammatory response | 1.40E-11 | 1.74E-08 |
| BP | GO:0022407 | regulation of cell-cell adhesion | 3.06E-09 | 1.36E-06 |
| BP | GO:0001819 | positive regulation of cytokine production | 4.08E-09 | 1.36E-06 |
| BP | GO:0030217 | T cell differentiation | 5.44E-09 | 1.36E-06 |
| BP | GO:0042110 | T cell activation | 5.46E-09 | 1.36E-06 |
| BP | GO:0031349 | positive regulation of defense response | 8.71E-09 | 1.76E-06 |
| BP | GO:0022409 | positive regulation of cell-cell adhesion | 9.89E-09 | 1.76E-06 |
| BP | GO:0002861 | regulation of inflammatory response to antigenic stimulus | 1.18E-08 | 1.83E-06 |
| BP | GO:0050729 | positive regulation of inflammatory response | 1.77E-08 | 2.45E-06 |
| BP | GO:0045058 | T cell selection | 2.19E-08 | 2.73E-06 |
| BP | GO:0030098 | lymphocyte differentiation | 5.10E-08 | 5.76E-06 |
| BP | GO:0050867 | positive regulation of cell activation | 1.01E-07 | 9.27E-06 |
| BP | GO:0002437 | inflammatory response to antigenic stimulus | 1.09E-07 | 9.27E-06 |
| BP | GO:1903131 | mononuclear cell differentiation | 1.10E-07 | 9.27E-06 |
| BP | GO:0032103 | positive regulation of response to external stimulus | 1.12E-07 | 9.27E-06 |
| BP | GO:0045785 | positive regulation of cell adhesion | 1.28E-07 | 9.97E-06 |
| BP | GO:1903039 | positive regulation of leukocyte cell-cell adhesion | 2.39E-07 | 1.75E-05 |
| BP | GO:0048872 | homeostasis of number of cells | 4.55E-07 | 3.14E-05 |
| BP | GO:0007204 | positive regulation of cytosolic calcium ion concentration | 9.99E-07 | 6.54E-05 |
| BP | GO:1903037 | regulation of leukocyte cell-cell adhesion | 1.29E-06 | 8.03E-05 |
| BP | GO:0032496 | response to lipopolysaccharide | 1.43E-06 | 8.46E-05 |
| BP | GO:0051480 | regulation of cytosolic calcium ion concentration | 1.65E-06 | 9.30E-05 |
| BP | GO:0051251 | positive regulation of lymphocyte activation | 1.86E-06 | 9.78E-05 |
| BP | GO:0002237 | response to molecule of bacterial origin | 1.89E-06 | 9.78E-05 |
| BP | GO:0007159 | leukocyte cell-cell adhesion | 2.10E-06 | 0.000104264 |
| BP | GO:0046631 | alpha-beta T cell activation | 2.18E-06 | 0.000104264 |
| BP | GO:0030099 | myeloid cell differentiation | 2.39E-06 | 0.000110272 |
| BP | GO:0031295 | T cell costimulation | 2.62E-06 | 0.00011662 |
| BP | GO:0031294 | lymphocyte costimulation | 3.01E-06 | 0.00012723 |
| BP | GO:0007259 | receptor signaling pathway via JAK-STAT | 3.07E-06 | 0.00012723 |
| BP | GO:0002696 | positive regulation of leukocyte activation | 3.39E-06 | 0.000135957 |
| BP | GO:0050728 | negative regulation of inflammatory response | 3.52E-06 | 0.000136914 |
| BP | GO:0097696 | receptor signaling pathway via STAT | 3.94E-06 | 0.000148394 |
| BP | GO:0006874 | cellular calcium ion homeostasis | 5.29E-06 | 0.000193475 |
| BP | GO:0055074 | calcium ion homeostasis | 6.02E-06 | 0.000213815 |
| BP | GO:0019221 | cytokine-mediated signaling pathway | 6.82E-06 | 0.000235676 |
| BP | GO:0072503 | cellular divalent inorganic cation homeostasis | 7.86E-06 | 0.000252988 |
| BP | GO:0009612 | response to mechanical stimulus | 7.93E-06 | 0.000252988 |
| BP | GO:0050870 | positive regulation of T cell activation | 7.93E-06 | 0.000252988 |
| BP | GO:0050851 | antigen receptor-mediated signaling pathway | 1.20E-05 | 0.000373908 |
| BP | GO:0033077 | T cell differentiation in thymus | 1.32E-05 | 0.000401545 |
| BP | GO:0043122 | regulation of I-kappaB kinase/NF-kappaB signaling | 1.39E-05 | 0.000411697 |
| BP | GO:0014068 | positive regulation of phosphatidylinositol 3-kinase signaling | 1.55E-05 | 0.000447732 |
| BP | GO:0031348 | negative regulation of defense response | 1.60E-05 | 0.000451936 |
| BP | GO:0045060 | negative thymic T cell selection | 2.06E-05 | 0.000559369 |
| BP | GO:1900182 | positive regulation of protein localization to nucleus | 2.07E-05 | 0.000559369 |
| BP | GO:0007249 | I-kappaB kinase/NF-kappaB signaling | 2.24E-05 | 0.000591793 |
| BP | GO:0034599 | cellular response to oxidative stress | 2.46E-05 | 0.000613528 |
| BP | GO:0002863 | positive regulation of inflammatory response to antigenic stimulus | 2.48E-05 | 0.000613528 |
| BP | GO:0043383 | negative T cell selection | 2.48E-05 | 0.000613528 |
| BP | GO:0002429 | immune response-activating cell surface receptor signaling pathway | 2.56E-05 | 0.000613528 |
| BP | GO:0002757 | immune response-activating signal transduction | 2.56E-05 | 0.000613528 |
| BP | GO:0036473 | cell death in response to oxidative stress | 2.69E-05 | 0.000632154 |
| BP | GO:0045639 | positive regulation of myeloid cell differentiation | 3.43E-05 | 0.00079045 |
| BP | GO:0002768 | immune response-regulating cell surface receptor signaling pathway | 3.50E-05 | 0.000791109 |
| BP | GO:0009895 | negative regulation of catabolic process | 3.72E-05 | 0.000826322 |
| BP | GO:0050863 | regulation of T cell activation | 4.15E-05 | 0.000904734 |
| BP | GO:0014066 | regulation of phosphatidylinositol 3-kinase signaling | 4.29E-05 | 0.000920382 |
| BP | GO:0046632 | alpha-beta T cell differentiation | 4.41E-05 | 0.000929349 |
| BP | GO:0062197 | cellular response to chemical stress | 4.55E-05 | 0.000943996 |
| BP | GO:0030278 | regulation of ossification | 4.77E-05 | 0.000972697 |
| BP | GO:0030218 | erythrocyte differentiation | 5.42E-05 | 0.001086576 |
| BP | GO:0050852 | T cell receptor signaling pathway | 5.83E-05 | 0.001151013 |
| BP | GO:1903706 | regulation of hemopoiesis | 6.35E-05 | 0.00123373 |
| BP | GO:0034101 | erythrocyte homeostasis | 6.72E-05 | 0.001285638 |
| BP | GO:0002253 | activation of immune response | 6.90E-05 | 0.001300973 |
| BP | GO:1900180 | regulation of protein localization to nucleus | 7.86E-05 | 0.001459595 |
| BP | GO:0045061 | thymic T cell selection | 8.64E-05 | 0.001580069 |
| BP | GO:0014065 | phosphatidylinositol 3-kinase signaling | 9.32E-05 | 0.001666191 |
| BP | GO:0034695 | response to prostaglandin E | 9.46E-05 | 0.001666191 |
| BP | GO:0043524 | negative regulation of neuron apoptotic process | 9.51E-05 | 0.001666191 |
| BP | GO:0042542 | response to hydrogen peroxide | 9.71E-05 | 0.001676912 |
| BP | GO:1903900 | regulation of viral life cycle | 0.000101055 | 0.001722085 |
| BP | GO:0032102 | negative regulation of response to external stimulus | 0.000107115 | 0.001800691 |
| BP | GO:0006816 | calcium ion transport | 0.000109102 | 0.001809636 |
| BP | GO:0002262 | myeloid cell homeostasis | 0.000120384 | 0.0019449 |
| BP | GO:2000377 | regulation of reactive oxygen species metabolic process | 0.000120384 | 0.0019449 |
| BP | GO:0060402 | calcium ion transport into cytosol | 0.000127325 | 0.00200497 |
| BP | GO:0090316 | positive regulation of intracellular protein transport | 0.000127325 | 0.00200497 |
| BP | GO:0007015 | actin filament organization | 0.00013048 | 0.002028963 |
| BP | GO:0006979 | response to oxidative stress | 0.000135097 | 0.002074821 |
| BP | GO:0050792 | regulation of viral process | 0.00013698 | 0.002078093 |
| BP | GO:0002862 | negative regulation of inflammatory response to antigenic stimulus | 0.000151424 | 0.002269543 |
| BP | GO:0034694 | response to prostaglandin | 0.000162183 | 0.002380427 |
| BP | GO:0002764 | immune response-regulating signaling pathway | 0.00016265 | 0.002380427 |
| BP | GO:0048015 | phosphatidylinositol-mediated signaling | 0.000174509 | 0.002524297 |
| BP | GO:0048017 | inositol lipid-mediated signaling | 0.00018634 | 0.002634172 |
| BP | GO:0060401 | cytosolic calcium ion transport | 0.00018634 | 0.002634172 |
| BP | GO:0045648 | positive regulation of erythrocyte differentiation | 0.000196646 | 0.002686522 |
| BP | GO:1901099 | negative regulation of signal transduction in absence of ligand | 0.000196646 | 0.002686522 |
| BP | GO:2001240 | negative regulation of extrinsic apoptotic signaling pathway in absence of ligand | 0.000196646 | 0.002686522 |
| BP | GO:0043123 | positive regulation of I-kappaB kinase/NF-kappaB signaling | 0.000198682 | 0.002686522 |
| BP | GO:0032743 | positive regulation of interleukin-2 production | 0.000208862 | 0.002793811 |
| BP | GO:0050731 | positive regulation of peptidyl-tyrosine phosphorylation | 0.000221537 | 0.002931836 |
| BP | GO:0002285 | lymphocyte activation involved in immune response | 0.000224936 | 0.002945474 |
| BP | GO:0032388 | positive regulation of intracellular transport | 0.000253348 | 0.003282972 |
| BP | GO:0002573 | myeloid leukocyte differentiation | 0.00027612 | 0.003505037 |
| BP | GO:1901215 | negative regulation of neuron death | 0.00027612 | 0.003505037 |
| BP | GO:0045637 | regulation of myeloid cell differentiation | 0.000283996 | 0.003568593 |
| BP | GO:0050807 | regulation of synapse organization | 0.000287988 | 0.003582566 |
| BP | GO:0043523 | regulation of neuron apoptotic process | 0.000292016 | 0.003596708 |
| BP | GO:0032735 | positive regulation of interleukin-12 production | 0.000304529 | 0.003677995 |
| BP | GO:0042307 | positive regulation of protein import into nucleus | 0.000304529 | 0.003677995 |
| BP | GO:0050803 | regulation of synapse structure or activity | 0.000316953 | 0.003791248 |
| BP | GO:0097191 | extrinsic apoptotic signaling pathway | 0.000321239 | 0.003805917 |
| BP | GO:0010810 | regulation of cell-substrate adhesion | 0.000329923 | 0.003824604 |
| BP | GO:0000302 | response to reactive oxygen species | 0.000334321 | 0.003824604 |
| BP | GO:1904591 | positive regulation of protein import | 0.000335114 | 0.003824604 |
| BP | GO:2001239 | regulation of extrinsic apoptotic signaling pathway in absence of ligand | 0.000335114 | 0.003824604 |
| BP | GO:0046427 | positive regulation of receptor signaling pathway via JAK-STAT | 0.000350947 | 0.00396889 |
| BP | GO:0033157 | regulation of intracellular protein transport | 0.000366177 | 0.00404178 |
| BP | GO:0071356 | cellular response to tumor necrosis factor | 0.000366177 | 0.00404178 |
| BP | GO:0008631 | intrinsic apoptotic signaling pathway in response to oxidative stress | 0.000367139 | 0.00404178 |
| BP | GO:0010830 | regulation of myotube differentiation | 0.000383691 | 0.00418694 |
| BP | GO:0045646 | regulation of erythrocyte differentiation | 0.000400601 | 0.004333457 |
| BP | GO:0051403 | stress-activated MAPK cascade | 0.000414998 | 0.004412454 |
| BP | GO:0072593 | reactive oxygen species metabolic process | 0.000414998 | 0.004412454 |
| BP | GO:1904894 | positive regulation of receptor signaling pathway via STAT | 0.000435496 | 0.004591162 |
| BP | GO:0051402 | neuron apoptotic process | 0.000451557 | 0.004720475 |
| BP | GO:0031098 | stress-activated protein kinase signaling cascade | 0.000456943 | 0.004736977 |
| BP | GO:0034612 | response to tumor necrosis factor | 0.000490134 | 0.004921021 |
| BP | GO:0097305 | response to alcohol | 0.000490134 | 0.004921021 |
| BP | GO:0031103 | axon regeneration | 0.00049052 | 0.004921021 |
| BP | GO:2000378 | negative regulation of reactive oxygen species metabolic process | 0.00049052 | 0.004921021 |
| BP | GO:0051924 | regulation of calcium ion transport | 0.000501533 | 0.004991259 |
| BP | GO:0031330 | negative regulation of cellular catabolic process | 0.000542774 | 0.005358816 |
| BP | GO:0050730 | regulation of peptidyl-tyrosine phosphorylation | 0.000554945 | 0.005435841 |
| BP | GO:0051155 | positive regulation of striated muscle cell differentiation | 0.000589349 | 0.005727737 |
| BP | GO:0010559 | regulation of glycoprotein biosynthetic process | 0.00061018 | 0.005884217 |
| BP | GO:0002366 | leukocyte activation involved in immune response | 0.000625034 | 0.005953046 |
| BP | GO:0048010 | vascular endothelial growth factor receptor signaling pathway | 0.000631365 | 0.005953046 |
| BP | GO:1903829 | positive regulation of cellular protein localization | 0.000631674 | 0.005953046 |
| BP | GO:0002263 | cell activation involved in immune response | 0.000651864 | 0.006016386 |
| BP | GO:0031102 | neuron projection regeneration | 0.000652904 | 0.006016386 |
| BP | GO:0042306 | regulation of protein import into nucleus | 0.000652904 | 0.006016386 |
| BP | GO:0032615 | interleukin-12 production | 0.000697041 | 0.006193704 |
| BP | GO:0032623 | interleukin-2 production | 0.000697041 | 0.006193704 |
| BP | GO:0032655 | regulation of interleukin-12 production | 0.000697041 | 0.006193704 |
| BP | GO:0032663 | regulation of interleukin-2 production | 0.000697041 | 0.006193704 |
| BP | GO:0046824 | positive regulation of nucleocytoplasmic transport | 0.000697041 | 0.006193704 |
| BP | GO:0046651 | lymphocyte proliferation | 0.000714911 | 0.006304436 |
| BP | GO:1904589 | regulation of protein import | 0.000719638 | 0.006304436 |
| BP | GO:0034504 | protein localization to nucleus | 0.000729432 | 0.006345549 |
| BP | GO:0032943 | mononuclear cell proliferation | 0.000736763 | 0.006364816 |
| BP | GO:1903018 | regulation of glycoprotein metabolic process | 0.000742588 | 0.006370891 |
| BP | GO:0038034 | signal transduction in absence of ligand | 0.000765889 | 0.00643761 |
| BP | GO:0048524 | positive regulation of viral process | 0.000765889 | 0.00643761 |
| BP | GO:0097192 | extrinsic apoptotic signaling pathway in absence of ligand | 0.000765889 | 0.00643761 |
| BP | GO:0042093 | T-helper cell differentiation | 0.000789542 | 0.006591882 |
| BP | GO:0051222 | positive regulation of protein transport | 0.000828449 | 0.006857554 |
| BP | GO:0002294 | CD4-positive, alpha-beta T cell differentiation involved in immune response | 0.000837901 | 0.006857554 |
| BP | GO:0042531 | positive regulation of tyrosine phosphorylation of STAT protein | 0.000837901 | 0.006857554 |
| BP | GO:0001666 | response to hypoxia | 0.000860556 | 0.006923103 |
| BP | GO:0002287 | alpha-beta T cell activation involved in immune response | 0.000862605 | 0.006923103 |
| BP | GO:0002293 | alpha-beta T cell differentiation involved in immune response | 0.000862605 | 0.006923103 |
| BP | GO:0006959 | humoral immune response | 0.000944282 | 0.00747665 |
| BP | GO:0019058 | viral life cycle | 0.000944282 | 0.00747665 |
| BP | GO:0070661 | leukocyte proliferation | 0.000952929 | 0.00747665 |
| BP | GO:1901214 | regulation of neuron death | 0.000961627 | 0.00747665 |
| BP | GO:1904951 | positive regulation of establishment of protein localization | 0.000961627 | 0.00747665 |
| BP | GO:0036293 | response to decreased oxygen levels | 0.000988025 | 0.00763418 |
| BP | GO:0002292 | T cell differentiation involved in immune response | 0.001018168 | 0.007818522 |
| BP | GO:0006809 | nitric oxide biosynthetic process | 0.001045313 | 0.007929085 |
| BP | GO:0014855 | striated muscle cell proliferation | 0.001045313 | 0.007929085 |
| BP | GO:0070371 | ERK1 and ERK2 cascade | 0.00106067 | 0.00799681 |
| BP | GO:0032386 | regulation of intracellular transport | 0.001126958 | 0.008445396 |
| BP | GO:0046209 | nitric oxide metabolic process | 0.001186243 | 0.008783845 |
| BP | GO:0051149 | positive regulation of muscle cell differentiation | 0.001186243 | 0.008783845 |
| BP | GO:2001057 | reactive nitrogen species metabolic process | 0.001215466 | 0.008946981 |
| BP | GO:0070482 | response to oxygen levels | 0.001226147 | 0.008972511 |
| BP | GO:0043367 | CD4-positive, alpha-beta T cell differentiation | 0.001245034 | 0.009004782 |
| BP | GO:0048678 | response to axon injury | 0.001245034 | 0.009004782 |
| BP | GO:0010507 | negative regulation of autophagy | 0.001305204 | 0.009278134 |
| BP | GO:0042509 | regulation of tyrosine phosphorylation of STAT protein | 0.001305204 | 0.009278134 |
| BP | GO:0045069 | regulation of viral genome replication | 0.001305204 | 0.009278134 |
| BP | GO:0002460 | adaptive immune response based on somatic recombination of immune receptors built from immunoglobulin superfamily domains | 0.001320022 | 0.009330152 |
| BP | GO:0070997 | neuron death | 0.001374091 | 0.009657451 |
| BP | GO:0031589 | cell-substrate adhesion | 0.001396106 | 0.009715955 |
| BP | GO:0007260 | tyrosine phosphorylation of STAT protein | 0.001398035 | 0.009715955 |
| BP | GO:0050900 | leukocyte migration | 0.001463492 | 0.010114355 |
| BP | GO:0018108 | peptidyl-tyrosine phosphorylation | 0.001532907 | 0.010535559 |
| BP | GO:0018212 | peptidyl-tyrosine modification | 0.001568383 | 0.010720154 |
| BP | GO:0042692 | muscle cell differentiation | 0.001640885 | 0.011154434 |
| BP | GO:0045860 | positive regulation of protein kinase activity | 0.001665516 | 0.011260334 |
| BP | GO:2001237 | negative regulation of extrinsic apoptotic signaling pathway | 0.001694992 | 0.011397676 |
| BP | GO:0051153 | regulation of striated muscle cell differentiation | 0.001764724 | 0.011802776 |
| BP | GO:0010522 | regulation of calcium ion transport into cytosol | 0.001871859 | 0.012386132 |
| BP | GO:0035710 | CD4-positive, alpha-beta T cell activation | 0.001871859 | 0.012386132 |
| BP | GO:0046425 | regulation of receptor signaling pathway via JAK-STAT | 0.001908246 | 0.012560097 |
| BP | GO:0010959 | regulation of metal ion transport | 0.001924763 | 0.012602132 |
| BP | GO:0001503 | ossification | 0.001952 | 0.012713552 |
| BP | GO:0042116 | macrophage activation | 0.002019428 | 0.013016417 |
| BP | GO:0046822 | regulation of nucleocytoplasmic transport | 0.002019428 | 0.013016417 |
| BP | GO:0043434 | response to peptide hormone | 0.002035168 | 0.013050253 |
| BP | GO:0016032 | viral process | 0.002049243 | 0.013056674 |
| BP | GO:0002824 | positive regulation of adaptive immune response based on somatic recombination of immune receptors built from immunoglobulin superfamily domains | 0.002057161 | 0.013056674 |
| BP | GO:0022037 | metencephalon development | 0.002095229 | 0.013230787 |
| BP | GO:0042060 | wound healing | 0.002149484 | 0.013436974 |
| BP | GO:0072594 | establishment of protein localization to organelle | 0.002149484 | 0.013436974 |
| BP | GO:0032611 | interleukin-1 beta production | 0.002172371 | 0.013444923 |
| BP | GO:0032651 | regulation of interleukin-1 beta production | 0.002172371 | 0.013444923 |
| BP | GO:0050808 | synapse organization | 0.002208124 | 0.013598546 |
| BP | GO:0002821 | positive regulation of adaptive immune response | 0.00225085 | 0.013793388 |
| BP | GO:0002683 | negative regulation of immune system process | 0.002328401 | 0.014074496 |
| BP | GO:0002286 | T cell activation involved in immune response | 0.002330664 | 0.014074496 |
| BP | GO:1904892 | regulation of receptor signaling pathway via STAT | 0.002330664 | 0.014074496 |
| BP | GO:0048732 | gland development | 0.0023591 | 0.014177391 |
| BP | GO:0014902 | myotube differentiation | 0.002452881 | 0.014670115 |
| BP | GO:0051897 | positive regulation of protein kinase B signaling | 0.002578084 | 0.01534515 |
| BP | GO:0010811 | positive regulation of cell-substrate adhesion | 0.002706263 | 0.015955409 |
| BP | GO:0030168 | platelet activation | 0.002706263 | 0.015955409 |
| BP | GO:0009410 | response to xenobiotic stimulus | 0.002781485 | 0.016321547 |
| BP | GO:0033674 | positive regulation of kinase activity | 0.002867756 | 0.016670508 |
| BP | GO:0061564 | axon development | 0.002867756 | 0.016670508 |
| BP | GO:0001952 | regulation of cell-matrix adhesion | 0.002926482 | 0.016776697 |
| BP | GO:0032612 | interleukin-1 production | 0.002926482 | 0.016776697 |
| BP | GO:0032652 | regulation of interleukin-1 production | 0.002926482 | 0.016776697 |
| BP | GO:0019079 | viral genome replication | 0.003062549 | 0.017476196 |
| BP | GO:0007569 | cell aging | 0.003108558 | 0.017657747 |
| BP | GO:0050671 | positive regulation of lymphocyte proliferation | 0.003343493 | 0.018905931 |
| BP | GO:0032946 | positive regulation of mononuclear cell proliferation | 0.003391454 | 0.019090358 |
| BP | GO:0045598 | regulation of fat cell differentiation | 0.00343974 | 0.01927494 |
| BP | GO:0097553 | calcium ion transmembrane import into cytosol | 0.003586538 | 0.020007415 |
| BP | GO:0070555 | response to interleukin-1 | 0.003636117 | 0.020193434 |
| BP | GO:0010212 | response to ionizing radiation | 0.003888836 | 0.021311507 |
| BP | GO:0097530 | granulocyte migration | 0.003888836 | 0.021311507 |
| BP | GO:2001056 | positive regulation of cysteine-type endopeptidase activity | 0.003888836 | 0.021311507 |
| BP | GO:0070665 | positive regulation of leukocyte proliferation | 0.00399217 | 0.02177978 |
| BP | GO:0030902 | hindbrain development | 0.004044316 | 0.02177978 |
| BP | GO:0071456 | cellular response to hypoxia | 0.004044316 | 0.02177978 |
| BP | GO:2001236 | regulation of extrinsic apoptotic signaling pathway | 0.004044316 | 0.02177978 |
| BP | GO:0050871 | positive regulation of B cell activation | 0.004096782 | 0.021967228 |
| BP | GO:0006606 | protein import into nucleus | 0.004256092 | 0.022530123 |
| BP | GO:0007519 | skeletal muscle tissue development | 0.004256092 | 0.022530123 |
| BP | GO:0051147 | regulation of muscle cell differentiation | 0.004256092 | 0.022530123 |
| BP | GO:0051170 | import into nucleus | 0.004472956 | 0.023577784 |
| BP | GO:0036294 | cellular response to decreased oxygen levels | 0.004583288 | 0.024057426 |
| BP | GO:0060538 | skeletal muscle organ development | 0.004864638 | 0.025426932 |
| BP | GO:0007254 | JNK cascade | 0.004921852 | 0.02561834 |
| BP | GO:0002822 | regulation of adaptive immune response based on somatic recombination of immune receptors built from immunoglobulin superfamily domains | 0.004979379 | 0.025809779 |
| BP | GO:0048639 | positive regulation of developmental growth | 0.005331108 | 0.027518249 |
| BP | GO:0071453 | cellular response to oxygen levels | 0.005511178 | 0.028330186 |
| BP | GO:0035265 | organ growth | 0.005571822 | 0.028524058 |
| BP | GO:0010950 | positive regulation of endopeptidase activity | 0.005632775 | 0.02871792 |
| BP | GO:0002819 | regulation of adaptive immune response | 0.00587968 | 0.029854377 |
| BP | GO:0051896 | regulation of protein kinase B signaling | 0.006004982 | 0.030366658 |
| BP | GO:0030041 | actin filament polymerization | 0.006388251 | 0.03106262 |
| BP | GO:0002524 | hypersensitivity | 0.006392308 | 0.03106262 |
| BP | GO:0010728 | regulation of hydrogen peroxide biosynthetic process | 0.006392308 | 0.03106262 |
| BP | GO:0014041 | regulation of neuron maturation | 0.006392308 | 0.03106262 |
| BP | GO:0021548 | pons development | 0.006392308 | 0.03106262 |
| BP | GO:0045348 | positive regulation of MHC class II biosynthetic process | 0.006392308 | 0.03106262 |
| BP | GO:0070391 | response to lipoteichoic acid | 0.006392308 | 0.03106262 |
| BP | GO:0071223 | cellular response to lipoteichoic acid | 0.006392308 | 0.03106262 |
| BP | GO:2000510 | positive regulation of dendritic cell chemotaxis | 0.006392308 | 0.03106262 |
| BP | GO:2001269 | positive regulation of cysteine-type endopeptidase activity involved in apoptotic signaling pathway | 0.006392308 | 0.03106262 |
| BP | GO:0007565 | female pregnancy | 0.006518452 | 0.031552351 |
| BP | GO:0050777 | negative regulation of immune response | 0.006584009 | 0.031623582 |
| BP | GO:1901654 | response to ketone | 0.006584009 | 0.031623582 |
| BP | GO:0006006 | glucose metabolic process | 0.006716036 | 0.031798784 |
| BP | GO:0010952 | positive regulation of peptidase activity | 0.006782505 | 0.031798784 |
| BP | GO:0031099 | regeneration | 0.006849277 | 0.031798784 |
| BP | GO:0050864 | regulation of B cell activation | 0.006849277 | 0.031798784 |
| BP | GO:0042098 | T cell proliferation | 0.006916351 | 0.031798784 |
| BP | GO:0002604 | regulation of dendritic cell antigen processing and presentation | 0.007029474 | 0.031798784 |
| BP | GO:0019048 | modulation by virus of host process | 0.007029474 | 0.031798784 |
| BP | GO:0021702 | cerebellar Purkinje cell differentiation | 0.007029474 | 0.031798784 |
| BP | GO:0033033 | negative regulation of myeloid cell apoptotic process | 0.007029474 | 0.031798784 |
| BP | GO:0044068 | modulation by symbiont of host cellular process | 0.007029474 | 0.031798784 |
| BP | GO:0045657 | positive regulation of monocyte differentiation | 0.007029474 | 0.031798784 |
| BP | GO:0046598 | positive regulation of viral entry into host cell | 0.007029474 | 0.031798784 |
| BP | GO:0075294 | positive regulation by symbiont of entry into host | 0.007029474 | 0.031798784 |
| BP | GO:0097048 | dendritic cell apoptotic process | 0.007029474 | 0.031798784 |
| BP | GO:1902065 | response to L-glutamate | 0.007029474 | 0.031798784 |
| BP | GO:2000668 | regulation of dendritic cell apoptotic process | 0.007029474 | 0.031798784 |
| BP | GO:0019722 | calcium-mediated signaling | 0.007119388 | 0.032088835 |
| BP | GO:0060348 | bone development | 0.007325136 | 0.032897001 |
| BP | GO:0017038 | protein import | 0.00739432 | 0.033088252 |
| BP | GO:0010623 | programmed cell death involved in cell development | 0.007666266 | 0.033733284 |
| BP | GO:0048742 | regulation of skeletal muscle fiber development | 0.007666266 | 0.033733284 |
| BP | GO:1902950 | regulation of dendritic spine maintenance | 0.007666266 | 0.033733284 |
| BP | GO:2000508 | regulation of dendritic cell chemotaxis | 0.007666266 | 0.033733284 |
| BP | GO:0002685 | regulation of leukocyte migration | 0.007674051 | 0.033733284 |
| BP | GO:0043491 | protein kinase B signaling | 0.007744731 | 0.033924104 |
| BP | GO:0051651 | maintenance of location in cell | 0.007958559 | 0.034543604 |
| BP | GO:0007596 | blood coagulation | 0.008175062 | 0.034543604 |
| BP | GO:0070374 | positive regulation of ERK1 and ERK2 cascade | 0.008175062 | 0.034543604 |
| BP | GO:0008154 | actin polymerization or depolymerization | 0.008247822 | 0.034543604 |
| BP | GO:0001768 | establishment of T cell polarity | 0.008302683 | 0.034543604 |
| BP | GO:0014842 | regulation of skeletal muscle satellite cell proliferation | 0.008302683 | 0.034543604 |
| BP | GO:0021694 | cerebellar Purkinje cell layer formation | 0.008302683 | 0.034543604 |
| BP | GO:0031272 | regulation of pseudopodium assembly | 0.008302683 | 0.034543604 |
| BP | GO:0031274 | positive regulation of pseudopodium assembly | 0.008302683 | 0.034543604 |
| BP | GO:0033689 | negative regulation of osteoblast proliferation | 0.008302683 | 0.034543604 |
| BP | GO:0034112 | positive regulation of homotypic cell-cell adhesion | 0.008302683 | 0.034543604 |
| BP | GO:0035331 | negative regulation of hippo signaling | 0.008302683 | 0.034543604 |
| BP | GO:0048070 | regulation of developmental pigmentation | 0.008302683 | 0.034543604 |
| BP | GO:0048302 | regulation of isotype switching to IgG isotypes | 0.008302683 | 0.034543604 |
| BP | GO:0120305 | regulation of pigmentation | 0.008302683 | 0.034543604 |
| BP | GO:0044706 | multi-multicellular organism process | 0.008394229 | 0.03469243 |
| BP | GO:0097529 | myeloid leukocyte migration | 0.008394229 | 0.03469243 |
| BP | GO:0007599 | hemostasis | 0.008541817 | 0.035069376 |
| BP | GO:0050817 | coagulation | 0.008541817 | 0.035069376 |
| BP | GO:0002274 | myeloid leukocyte activation | 0.008616053 | 0.035078156 |
| BP | GO:2001234 | negative regulation of apoptotic signaling pathway | 0.008690583 | 0.035078156 |
| BP | GO:0050670 | regulation of lymphocyte proliferation | 0.008765407 | 0.035078156 |
| BP | GO:0032944 | regulation of mononuclear cell proliferation | 0.008915934 | 0.035078156 |
| BP | GO:0046777 | protein autophosphorylation | 0.008915934 | 0.035078156 |
| BP | GO:0098657 | import into cell | 0.008915934 | 0.035078156 |
| BP | GO:0001767 | establishment of lymphocyte polarity | 0.008938726 | 0.035078156 |
| BP | GO:0001771 | immunological synapse formation | 0.008938726 | 0.035078156 |
| BP | GO:0002864 | regulation of acute inflammatory response to antigenic stimulus | 0.008938726 | 0.035078156 |
| BP | GO:0034616 | response to laminar fluid shear stress | 0.008938726 | 0.035078156 |
| BP | GO:0043374 | CD8-positive, alpha-beta T cell differentiation | 0.008938726 | 0.035078156 |
| BP | GO:0048291 | isotype switching to IgG isotypes | 0.008938726 | 0.035078156 |
| BP | GO:0050966 | detection of mechanical stimulus involved in sensory perception of pain | 0.008938726 | 0.035078156 |
| BP | GO:1902166 | negative regulation of intrinsic apoptotic signaling pathway in response to DNA damage by p53 class mediator | 0.008938726 | 0.035078156 |
| BP | GO:0045444 | fat cell differentiation | 0.009067632 | 0.035472121 |
| BP | GO:0007160 | cell-matrix adhesion | 0.00937453 | 0.036202273 |
| BP | GO:2000116 | regulation of cysteine-type endopeptidase activity | 0.009529725 | 0.036202273 |
| BP | GO:0002468 | dendritic cell antigen processing and presentation | 0.009574395 | 0.036202273 |
| BP | GO:0014841 | skeletal muscle satellite cell proliferation | 0.009574395 | 0.036202273 |
| BP | GO:0014857 | regulation of skeletal muscle cell proliferation | 0.009574395 | 0.036202273 |
| BP | GO:0031269 | pseudopodium assembly | 0.009574395 | 0.036202273 |
| BP | GO:0031958 | corticosteroid receptor signaling pathway | 0.009574395 | 0.036202273 |
| BP | GO:0042976 | activation of Janus kinase activity | 0.009574395 | 0.036202273 |
| BP | GO:0045346 | regulation of MHC class II biosynthetic process | 0.009574395 | 0.036202273 |
| BP | GO:0071380 | cellular response to prostaglandin E stimulus | 0.009574395 | 0.036202273 |
| BP | GO:1901741 | positive regulation of myoblast fusion | 0.009574395 | 0.036202273 |
| BP | GO:0019318 | hexose metabolic process | 0.009686079 | 0.036513583 |
| BP | GO:0030575 | nuclear body organization | 0.010209691 | 0.03746565 |
| BP | GO:0031268 | pseudopodium organization | 0.010209691 | 0.03746565 |
| BP | GO:0044849 | estrous cycle | 0.010209691 | 0.03746565 |
| BP | GO:0045342 | MHC class II biosynthetic process | 0.010209691 | 0.03746565 |
| BP | GO:0050862 | positive regulation of T cell receptor signaling pathway | 0.010209691 | 0.03746565 |
| BP | GO:0051770 | positive regulation of nitric-oxide synthase biosynthetic process | 0.010209691 | 0.03746565 |
| BP | GO:0090336 | positive regulation of brown fat cell differentiation | 0.010209691 | 0.03746565 |
| BP | GO:1902165 | regulation of intrinsic apoptotic signaling pathway in response to DNA damage by p53 class mediator | 0.010209691 | 0.03746565 |
| BP | GO:2001267 | regulation of cysteine-type endopeptidase activity involved in apoptotic signaling pathway | 0.010209691 | 0.03746565 |
| BP | GO:0070663 | regulation of leukocyte proliferation | 0.010323043 | 0.037770192 |
| BP | GO:0033002 | muscle cell proliferation | 0.010566643 | 0.038001967 |
| BP | GO:1901988 | negative regulation of cell cycle phase transition | 0.010648414 | 0.038001967 |
| BP | GO:0003015 | heart process | 0.010812813 | 0.038001967 |
| BP | GO:0010224 | response to UV-B | 0.010844613 | 0.038001967 |
| BP | GO:0010715 | regulation of extracellular matrix disassembly | 0.010844613 | 0.038001967 |
| BP | GO:0010831 | positive regulation of myotube differentiation | 0.010844613 | 0.038001967 |
| BP | GO:0014856 | skeletal muscle cell proliferation | 0.010844613 | 0.038001967 |
| BP | GO:0021692 | cerebellar Purkinje cell layer morphogenesis | 0.010844613 | 0.038001967 |
| BP | GO:0021924 | cell proliferation in external granule layer | 0.010844613 | 0.038001967 |
| BP | GO:0021930 | cerebellar granule cell precursor proliferation | 0.010844613 | 0.038001967 |
| BP | GO:0033623 | regulation of integrin activation | 0.010844613 | 0.038001967 |
| BP | GO:0038065 | collagen-activated signaling pathway | 0.010844613 | 0.038001967 |
| BP | GO:0042753 | positive regulation of circadian rhythm | 0.010844613 | 0.038001967 |
| BP | GO:0046068 | cGMP metabolic process | 0.010844613 | 0.038001967 |
| BP | GO:2000811 | negative regulation of anoikis | 0.010844613 | 0.038001967 |
| BP | GO:0005996 | monosaccharide metabolic process | 0.01131283 | 0.039239883 |
| BP | GO:0002223 | stimulatory C-type lectin receptor signaling pathway | 0.011479161 | 0.039239883 |
| BP | GO:0021534 | cell proliferation in hindbrain | 0.011479161 | 0.039239883 |
| BP | GO:0048643 | positive regulation of skeletal muscle tissue development | 0.011479161 | 0.039239883 |
| BP | GO:0050665 | hydrogen peroxide biosynthetic process | 0.011479161 | 0.039239883 |
| BP | GO:0060391 | positive regulation of SMAD protein signal transduction | 0.011479161 | 0.039239883 |
| BP | GO:1990840 | response to lectin | 0.011479161 | 0.039239883 |
| BP | GO:1990858 | cellular response to lectin | 0.011479161 | 0.039239883 |
| BP | GO:0045927 | positive regulation of growth | 0.011481767 | 0.039239883 |
| BP | GO:0030522 | intracellular receptor signaling pathway | 0.011995332 | 0.040837373 |
| BP | GO:0032695 | negative regulation of interleukin-12 production | 0.012113337 | 0.040837373 |
| BP | GO:0033194 | response to hydroperoxide | 0.012113337 | 0.040837373 |
| BP | GO:0044003 | modulation by symbiont of host process | 0.012113337 | 0.040837373 |
| BP | GO:1901739 | regulation of myoblast fusion | 0.012113337 | 0.040837373 |
| BP | GO:0002577 | regulation of antigen processing and presentation | 0.012747139 | 0.042062178 |
| BP | GO:0010523 | negative regulation of calcium ion transport into cytosol | 0.012747139 | 0.042062178 |
| BP | GO:0032495 | response to muramyl dipeptide | 0.012747139 | 0.042062178 |
| BP | GO:0045844 | positive regulation of striated muscle tissue development | 0.012747139 | 0.042062178 |
| BP | GO:0048636 | positive regulation of muscle organ development | 0.012747139 | 0.042062178 |
| BP | GO:0051900 | regulation of mitochondrial depolarization | 0.012747139 | 0.042062178 |
| BP | GO:0071379 | cellular response to prostaglandin stimulus | 0.012747139 | 0.042062178 |
| BP | GO:0071731 | response to nitric oxide | 0.012747139 | 0.042062178 |
| BP | GO:0110053 | regulation of actin filament organization | 0.013142537 | 0.042460344 |
| BP | GO:1902105 | regulation of leukocyte differentiation | 0.013232722 | 0.042460344 |
| BP | GO:0001502 | cartilage condensation | 0.013380569 | 0.042460344 |
| BP | GO:0021533 | cell differentiation in hindbrain | 0.013380569 | 0.042460344 |
| BP | GO:0032516 | positive regulation of phosphoprotein phosphatase activity | 0.013380569 | 0.042460344 |
| BP | GO:0035330 | regulation of hippo signaling | 0.013380569 | 0.042460344 |
| BP | GO:0045655 | regulation of monocyte differentiation | 0.013380569 | 0.042460344 |
| BP | GO:0046641 | positive regulation of alpha-beta T cell proliferation | 0.013380569 | 0.042460344 |
| BP | GO:0051767 | nitric-oxide synthase biosynthetic process | 0.013380569 | 0.042460344 |
| BP | GO:0051769 | regulation of nitric-oxide synthase biosynthetic process | 0.013380569 | 0.042460344 |
| BP | GO:0097062 | dendritic spine maintenance | 0.013380569 | 0.042460344 |
| BP | GO:1901863 | positive regulation of muscle tissue development | 0.013380569 | 0.042460344 |
| BP | GO:1902254 | negative regulation of intrinsic apoptotic signaling pathway by p53 class mediator | 0.013380569 | 0.042460344 |
| BP | GO:1903427 | negative regulation of reactive oxygen species biosynthetic process | 0.013380569 | 0.042460344 |
| BP | GO:1903429 | regulation of cell maturation | 0.013380569 | 0.042460344 |
| BP | GO:0006470 | protein dephosphorylation | 0.013413919 | 0.042460344 |
| BP | GO:0051146 | striated muscle cell differentiation | 0.013596214 | 0.042928148 |
| BP | GO:0002220 | innate immune response activating cell surface receptor signaling pathway | 0.014013626 | 0.043473692 |
| BP | GO:0002320 | lymphoid progenitor cell differentiation | 0.014013626 | 0.043473692 |
| BP | GO:0010310 | regulation of hydrogen peroxide metabolic process | 0.014013626 | 0.043473692 |
| BP | GO:0021854 | hypothalamus development | 0.014013626 | 0.043473692 |
| BP | GO:0031281 | positive regulation of cyclase activity | 0.014013626 | 0.043473692 |
| BP | GO:0036120 | cellular response to platelet-derived growth factor stimulus | 0.014013626 | 0.043473692 |
| BP | GO:0045663 | positive regulation of myoblast differentiation | 0.014013626 | 0.043473692 |
| BP | GO:0097193 | intrinsic apoptotic signaling pathway | 0.014056742 | 0.043498971 |
| BP | GO:0071375 | cellular response to peptide hormone stimulus | 0.014242862 | 0.043965558 |
| BP | GO:0010948 | negative regulation of cell cycle process | 0.01461836 | 0.044009686 |
| BP | GO:0002363 | alpha-beta T cell lineage commitment | 0.01464631 | 0.044009686 |
| BP | GO:0002758 | innate immune response-activating signal transduction | 0.01464631 | 0.044009686 |
| BP | GO:0035994 | response to muscle stretch | 0.01464631 | 0.044009686 |
| BP | GO:0036119 | response to platelet-derived growth factor | 0.01464631 | 0.044009686 |
| BP | GO:0043369 | CD4-positive or CD8-positive, alpha-beta T cell lineage commitment | 0.01464631 | 0.044009686 |
| BP | GO:0051882 | mitochondrial depolarization | 0.01464631 | 0.044009686 |
| BP | GO:0060143 | positive regulation of syncytium formation by plasma membrane fusion | 0.01464631 | 0.044009686 |
| BP | GO:0060396 | growth hormone receptor signaling pathway | 0.01464631 | 0.044009686 |
| BP | GO:0072677 | eosinophil migration | 0.01464631 | 0.044009686 |
| BP | GO:0090335 | regulation of brown fat cell differentiation | 0.01464631 | 0.044009686 |
| BP | GO:0051258 | protein polymerization | 0.014902825 | 0.044325495 |
| BP | GO:0048511 | rhythmic process | 0.014998186 | 0.044325495 |
| BP | GO:0002438 | acute inflammatory response to antigenic stimulus | 0.015278623 | 0.044325495 |
| BP | GO:0002922 | positive regulation of humoral immune response | 0.015278623 | 0.044325495 |
| BP | GO:0006582 | melanin metabolic process | 0.015278623 | 0.044325495 |
| BP | GO:0021697 | cerebellar cortex formation | 0.015278623 | 0.044325495 |
| BP | GO:0033032 | regulation of myeloid cell apoptotic process | 0.015278623 | 0.044325495 |
| BP | GO:0048641 | regulation of skeletal muscle tissue development | 0.015278623 | 0.044325495 |
| BP | GO:0070935 | 3'-UTR-mediated mRNA stabilization | 0.015278623 | 0.044325495 |
| BP | GO:0071378 | cellular response to growth hormone stimulus | 0.015278623 | 0.044325495 |
| BP | GO:0090023 | positive regulation of neutrophil chemotaxis | 0.015278623 | 0.044325495 |
| BP | GO:0098743 | cell aggregation | 0.015278623 | 0.044325495 |
| BP | GO:2000209 | regulation of anoikis | 0.015278623 | 0.044325495 |
| BP | GO:0006913 | nucleocytoplasmic transport | 0.015285882 | 0.044325495 |
| BP | GO:0051169 | nuclear transport | 0.015285882 | 0.044325495 |
| BP | GO:0010560 | positive regulation of glycoprotein biosynthetic process | 0.015910564 | 0.044881499 |
| BP | GO:0030318 | melanocyte differentiation | 0.015910564 | 0.044881499 |
| BP | GO:0032469 | endoplasmic reticulum calcium ion homeostasis | 0.015910564 | 0.044881499 |
| BP | GO:0032753 | positive regulation of interleukin-4 production | 0.015910564 | 0.044881499 |
| BP | GO:0033622 | integrin activation | 0.015910564 | 0.044881499 |
| BP | GO:0036315 | cellular response to sterol | 0.015910564 | 0.044881499 |
| BP | GO:0045822 | negative regulation of heart contraction | 0.015910564 | 0.044881499 |
| BP | GO:0062149 | detection of stimulus involved in sensory perception of pain | 0.015910564 | 0.044881499 |
| BP | GO:0090330 | regulation of platelet aggregation | 0.015910564 | 0.044881499 |
| BP | GO:0098703 | calcium ion import across plasma membrane | 0.015910564 | 0.044881499 |
| BP | GO:0099560 | synaptic membrane adhesion | 0.015910564 | 0.044881499 |
| BP | GO:1904996 | positive regulation of leukocyte adhesion to vascular endothelial cell | 0.015910564 | 0.044881499 |
| BP | GO:0070372 | regulation of ERK1 and ERK2 cascade | 0.016064849 | 0.04521419 |
| BP | GO:0019932 | second-messenger-mediated signaling | 0.016361353 | 0.045729806 |
| BP | GO:0070588 | calcium ion transmembrane transport | 0.016361353 | 0.045729806 |
| BP | GO:0002407 | dendritic cell chemotaxis | 0.016542132 | 0.045729806 |
| BP | GO:0021680 | cerebellar Purkinje cell layer development | 0.016542132 | 0.045729806 |
| BP | GO:0050857 | positive regulation of antigen receptor-mediated signaling pathway | 0.016542132 | 0.045729806 |
| BP | GO:0070102 | interleukin-6-mediated signaling pathway | 0.016542132 | 0.045729806 |
| BP | GO:1902656 | calcium ion import into cytosol | 0.016542132 | 0.045729806 |
| BP | GO:1903523 | negative regulation of blood circulation | 0.016542132 | 0.045729806 |
| BP | GO:0018105 | peptidyl-serine phosphorylation | 0.016660238 | 0.045954183 |
| BP | GO:0009101 | glycoprotein biosynthetic process | 0.016860815 | 0.046141732 |
| BP | GO:0010506 | regulation of autophagy | 0.016860815 | 0.046141732 |
| BP | GO:0071496 | cellular response to external stimulus | 0.017163651 | 0.046141732 |
| BP | GO:0002433 | immune response-regulating cell surface receptor signaling pathway involved in phagocytosis | 0.01717333 | 0.046141732 |
| BP | GO:0033688 | regulation of osteoblast proliferation | 0.01717333 | 0.046141732 |
| BP | GO:0036037 | CD8-positive, alpha-beta T cell activation | 0.01717333 | 0.046141732 |
| BP | GO:0038096 | Fc-gamma receptor signaling pathway involved in phagocytosis | 0.01717333 | 0.046141732 |
| BP | GO:0045830 | positive regulation of isotype switching | 0.01717333 | 0.046141732 |
| BP | GO:0048873 | homeostasis of number of cells within a tissue | 0.01717333 | 0.046141732 |
| BP | GO:0060142 | regulation of syncytium formation by plasma membrane fusion | 0.01717333 | 0.046141732 |
| BP | GO:0060333 | interferon-gamma-mediated signaling pathway | 0.01717333 | 0.046141732 |
| BP | GO:0071624 | positive regulation of granulocyte chemotaxis | 0.01717333 | 0.046141732 |
| BP | GO:0002675 | positive regulation of acute inflammatory response | 0.017804155 | 0.047222947 |
| BP | GO:0010575 | positive regulation of vascular endothelial growth factor production | 0.017804155 | 0.047222947 |
| BP | GO:0038094 | Fc-gamma receptor signaling pathway | 0.017804155 | 0.047222947 |
| BP | GO:0045589 | regulation of regulatory T cell differentiation | 0.017804155 | 0.047222947 |
| BP | GO:1902624 | positive regulation of neutrophil migration | 0.017804155 | 0.047222947 |
| BP | GO:1903020 | positive regulation of glycoprotein metabolic process | 0.017804155 | 0.047222947 |
| BP | GO:0007517 | muscle organ development | 0.017879428 | 0.047222947 |
| BP | GO:0051235 | maintenance of location | 0.017879428 | 0.047222947 |
| BP | GO:0048638 | regulation of developmental growth | 0.018190094 | 0.047381518 |
| BP | GO:0019216 | regulation of lipid metabolic process | 0.018294167 | 0.047381518 |
| BP | GO:0071214 | cellular response to abiotic stimulus | 0.018294167 | 0.047381518 |
| BP | GO:0104004 | cellular response to environmental stimulus | 0.018294167 | 0.047381518 |
| BP | GO:0002360 | T cell lineage commitment | 0.01843461 | 0.047381518 |
| BP | GO:0031069 | hair follicle morphogenesis | 0.01843461 | 0.047381518 |
| BP | GO:0033028 | myeloid cell apoptotic process | 0.01843461 | 0.047381518 |
| BP | GO:0040018 | positive regulation of multicellular organism growth | 0.01843461 | 0.047381518 |
| BP | GO:0048753 | pigment granule organization | 0.01843461 | 0.047381518 |
| BP | GO:0072539 | T-helper 17 cell differentiation | 0.01843461 | 0.047381518 |
| BP | GO:1902230 | negative regulation of intrinsic apoptotic signaling pathway in response to DNA damage | 0.01843461 | 0.047381518 |
| BP | GO:1902253 | regulation of intrinsic apoptotic signaling pathway by p53 class mediator | 0.01843461 | 0.047381518 |
| BP | GO:1903902 | positive regulation of viral life cycle | 0.01843461 | 0.047381518 |
| BP | GO:0042113 | B cell activation | 0.018607939 | 0.047728404 |
| BP | GO:0018209 | peptidyl-serine modification | 0.019029906 | 0.048483165 |
| BP | GO:0045070 | positive regulation of viral genome replication | 0.019064694 | 0.048483165 |
| BP | GO:0051491 | positive regulation of filopodium assembly | 0.019064694 | 0.048483165 |
| BP | GO:0090314 | positive regulation of protein targeting to membrane | 0.019064694 | 0.048483165 |
| BP | GO:0007568 | aging | 0.01913604 | 0.048483165 |
| BP | GO:0048545 | response to steroid hormone | 0.01913604 | 0.048483165 |
| BP | GO:0030336 | negative regulation of cell migration | 0.019670544 | 0.04919647 |
| BP | GO:0001782 | B cell homeostasis | 0.019694407 | 0.04919647 |
| BP | GO:0045066 | regulatory T cell differentiation | 0.019694407 | 0.04919647 |
| BP | GO:0060045 | positive regulation of cardiac muscle cell proliferation | 0.019694407 | 0.04919647 |
| BP | GO:0060055 | angiogenesis involved in wound healing | 0.019694407 | 0.04919647 |
| BP | GO:0060390 | regulation of SMAD protein signal transduction | 0.019694407 | 0.04919647 |
| BP | GO:1901797 | negative regulation of signal transduction by p53 class mediator | 0.019694407 | 0.04919647 |
| CC | GO:0045121 | membrane raft | 0.000978004 | 0.018063881 |
| CC | GO:0098857 | membrane microdomain | 0.000978004 | 0.018063881 |
| CC | GO:0098978 | glutamatergic synapse | 0.001003549 | 0.018063881 |
| CC | GO:0009897 | external side of plasma membrane | 0.001887415 | 0.025480097 |
| MF | GO:0004896 | cytokine receptor activity | 3.03E-05 | 0.002267158 |
| MF | GO:0051428 | peptide hormone receptor binding | 8.16E-05 | 0.002267158 |
| MF | GO:0019955 | cytokine binding | 8.88E-05 | 0.002267158 |
| MF | GO:0140375 | immune receptor activity | 9.86E-05 | 0.002267158 |
| MF | GO:0051427 | hormone receptor binding | 0.000204277 | 0.003326811 |
| MF | GO:0043548 | phosphatidylinositol 3-kinase binding | 0.000216966 | 0.003326811 |
| MF | GO:0004715 | non-membrane spanning protein tyrosine kinase activity | 0.000381355 | 0.004991471 |
| MF | GO:0015026 | coreceptor activity | 0.000434041 | 0.004991471 |
| MF | GO:0004714 | transmembrane receptor protein tyrosine kinase activity | 0.002854567 | 0.0267109 |
| MF | GO:0002020 | protease binding | 0.003372242 | 0.0267109 |
| MF | GO:0004713 | protein tyrosine kinase activity | 0.003421332 | 0.0267109 |
| MF | GO:0070851 | growth factor receptor binding | 0.003671825 | 0.0267109 |
| MF | GO:0019199 | transmembrane receptor protein kinase activity | 0.003774366 | 0.0267109 |
| MF | GO:0019903 | protein phosphatase binding | 0.004143757 | 0.027230405 |
| MF | GO:0004955 | prostaglandin receptor activity | 0.00651552 | 0.031255778 |
| MF | GO:0019902 | phosphatase binding | 0.006968849 | 0.031255778 |
| MF | GO:0004954 | prostanoid receptor activity | 0.007164927 | 0.031255778 |
| MF | GO:0042608 | T cell receptor binding | 0.007164927 | 0.031255778 |
| MF | GO:0043560 | insulin receptor substrate binding | 0.007164927 | 0.031255778 |
| MF | GO:0051400 | BH domain binding | 0.007164927 | 0.031255778 |
| MF | GO:0070513 | death domain binding | 0.007164927 | 0.031255778 |
| MF | GO:0033130 | acetylcholine receptor binding | 0.007813944 | 0.031255778 |
| MF | GO:0035256 | G protein-coupled glutamate receptor binding | 0.007813944 | 0.031255778 |
| MF | GO:0004707 | MAP kinase activity | 0.009110814 | 0.034924785 |
| MF | GO:0004953 | icosanoid receptor activity | 0.009758665 | 0.03545792 |
| MF | GO:0016004 | phospholipase activator activity | 0.010406129 | 0.03545792 |
| MF | GO:0035173 | histone kinase activity | 0.010406129 | 0.03545792 |
| MF | GO:0004708 | MAP kinase kinase activity | 0.011699891 | 0.037116897 |
| MF | GO:0060229 | lipase activator activity | 0.011699891 | 0.037116897 |
| MF | GO:0050321 | tau-protein kinase activity | 0.014282767 | 0.040817623 |
| MF | GO:0016493 | C-C chemokine receptor activity | 0.014927518 | 0.040817623 |
| MF | GO:0001664 | G protein-coupled receptor binding | 0.01525638 | 0.040817623 |
| MF | GO:0019957 | C-C chemokine binding | 0.015571883 | 0.040817623 |
| MF | GO:0043274 | phospholipase binding | 0.015571883 | 0.040817623 |
| MF | GO:0035035 | histone acetyltransferase binding | 0.016215861 | 0.040817623 |
| MF | GO:0051019 | mitogen-activated protein kinase binding | 0.016215861 | 0.040817623 |
| MF | GO:0001637 | G protein-coupled chemoattractant receptor activity | 0.016859453 | 0.040817623 |
| MF | GO:0004950 | chemokine receptor activity | 0.016859453 | 0.040817623 |
| MF | GO:0023026 | MHC class II protein complex binding | 0.017502659 | 0.041288324 |
| MF | GO:0046875 | ephrin receptor binding | 0.01814548 | 0.041734603 |
| MF | GO:0001222 | transcription corepressor binding | 0.020712908 | 0.044315988 |
| MF | GO:0001223 | transcription coactivator binding | 0.020712908 | 0.044315988 |
| MF | GO:0051721 | protein phosphatase 2A binding | 0.020712908 | 0.044315988 |
| MF | GO:0019956 | chemokine binding | 0.021353802 | 0.044648859 |
| MF | GO:0016248 | channel inhibitor activity | 0.021994312 | 0.04496615 |
| MF | GO:0023023 | MHC protein complex binding | 0.02327418 | 0.04555797 |
| MF | GO:0097718 | disordered domain specific binding | 0.02327418 | 0.04555797 |
| MF | GO:0042169 | SH2 domain binding | 0.025829311 | 0.047403607 |
| MF | GO:0042287 | MHC protein binding | 0.025829311 | 0.047403607 |
| MF | GO:0043014 | alpha-tubulin binding | 0.025829311 | 0.047403607 |
| MF | GO:0140297 | DNA-binding transcription factor binding | 0.026278086 | 0.047403607 |
