# Supplementary material for: Identification of immune-related lncRNA in sepsis by construction of ceRNA network and integrating bioinformatic analysis
Source: BMC Genomics. 2023 Aug 24;24:484. doi: 10.1186/s12864-023-09535-7 (PMC10464037; doi:10.1186/s12864-023-09535-7)
Supplement: Supplementary file 2 — Additional file 2: Supplementary Table S2. KEGG pathway enrichment assessments of IRDEGs regulated by lncRNAs in SPIMC network [file 12864_2023_9535_MOESM2_ESM.docx]

**Additional files:**

**Additional file 2:**

Supplementary Table 2: KEGG pathway enrichment assessments of IRDEGs regulated by lncRNAs in SPIMC network.

| ID | Description | pvalue | p.adjust |
| --- | --- | --- | --- |
| hsa05235 | PD-L1 expression and PD-1 checkpoint pathway in cancer | 6.91E-06 | 0.000368989 |
| hsa04658 | Th1 and Th2 cell differentiation | 7.88E-06 | 0.000368989 |
| hsa04660 | T cell receptor signaling pathway | 1.28E-05 | 0.000368989 |
| hsa04659 | Th17 cell differentiation | 1.44E-05 | 0.000368989 |
| hsa05145 | Toxoplasmosis | 1.72E-05 | 0.000368989 |
| hsa04630 | Jak-STAT signaling pathway | 7.37E-05 | 0.001126264 |
| hsa05161 | Hepatitis B | 7.37E-05 | 0.001126264 |
| hsa05152 | Tuberculosis | 0.000108695 | 0.001453792 |
| hsa04933 | AGE-RAGE signaling pathway in diabetic complications | 0.000396484 | 0.004713754 |
| hsa04725 | Cholinergic synapse | 0.000553133 | 0.005918526 |
| hsa04071 | Sphingolipid signaling pathway | 0.000660546 | 0.00628805 |
| hsa05200 | Pathways in cancer | 0.000705202 | 0.00628805 |
| hsa04217 | Necroptosis | 0.001617018 | 0.013309305 |
| hsa05014 | Amyotrophic lateral sclerosis (ALS) | 0.002578774 | 0.019709203 |
| hsa05170 | Human immunodeficiency virus 1 infection | 0.003486676 | 0.023760055 |
| hsa05416 | Viral myocarditis | 0.003552905 | 0.023760055 |
| hsa05163 | Human cytomegalovirus infection | 0.004125214 | 0.024714729 |
| hsa05321 | Inflammatory bowel disease (IBD) | 0.004157618 | 0.024714729 |
| hsa04664 | Fc epsilon RI signaling pathway | 0.0045419 | 0.025578067 |
| hsa04917 | Prolactin signaling pathway | 0.004806947 | 0.025717169 |
| hsa05140 | Leishmaniasis | 0.005644201 | 0.028758546 |
| hsa01521 | EGFR tyrosine kinase inhibitor resistance | 0.006086281 | 0.02960146 |
| hsa04060 | Cytokine-cytokine receptor interaction | 0.008698746 | 0.039536346 |
| hsa01522 | Endocrine resistance | 0.009240171 | 0.039536346 |
| hsa04061 | Viral protein interaction with cytokine and cytokine receptor | 0.009606963 | 0.039536346 |
| hsa04750 | Inflammatory mediator regulation of TRP channels | 0.009606963 | 0.039536346 |
| hsa04722 | Neurotrophin signaling pathway | 0.013410159 | 0.053143965 |
| hsa04611 | Platelet activation | 0.014504549 | 0.055428099 |
| hsa04380 | Osteoclast differentiation | 0.015407395 | 0.056847975 |
| hsa05162 | Measles | 0.017768912 | 0.061053 |
| hsa05418 | Fluid shear stress and atherosclerosis | 0.01801315 | 0.061053 |
| hsa04550 | Signaling pathways regulating pluripotency of stem cells | 0.018258841 | 0.061053 |
| hsa04514 | Cell adhesion molecules (CAMs) | 0.020019028 | 0.064624166 |
| hsa04261 | Adrenergic signaling in cardiomyocytes | 0.020534782 | 0.064624166 |
| hsa04218 | Cellular senescence | 0.023471505 | 0.071755743 |
| hsa04621 | NOD-like receptor signaling pathway | 0.029532887 | 0.087778304 |
| hsa05167 | Kaposi sarcoma-associated herpesvirus infection | 0.031060898 | 0.089824758 |
| hsa04062 | Chemokine signaling pathway | 0.031992912 | 0.090085304 |
| hsa04510 | Focal adhesion | 0.035180511 | 0.093514701 |
| hsa05130 | Pathogenic Escherichia coli infection | 0.035832736 | 0.093514701 |
| hsa05169 | Epstein-Barr virus infection | 0.035832736 | 0.093514701 |
