# Supplementary material for: Identification of immune-related lncRNA in sepsis by construction of ceRNA network and integrating bioinformatic analysis
Source: BMC Genomics. 2023 Aug 24;24:484. doi: 10.1186/s12864-023-09535-7 (PMC10464037; doi:10.1186/s12864-023-09535-7)
Supplement: Supplementary file 3 — Additional file 3: Supplementary Table S3. Differentially expressed lncRNAs in the GSE217700 dataset [file 12864_2023_9535_MOESM3_ESM.docx]

**Additional files:**

**Additional file 3:**

Supplementary Table S3: Differentially expressed lncRNAs in the GSE217700 dataset

| LncRNA | log2FoldChange | pvalue |
| --- | --- | --- |
| NEAT1 | 6.3839983 | 2.22E-127 |
| LINC02656 | 6.8037498 | 3.43E-109 |
| LINC02596 | 7.0267158 | 2.39E-82 |
| LINC01270 | 5.200155 | 9.01E-77 |
| LINC01001 | 4.9883362 | 1.14E-72 |
| LINC01271 | 5.1136226 | 6.21E-69 |
| FAM157B | 4.8430774 | 6.94E-65 |
| LINC02649 | 6.1338619 | 2.62E-62 |
| BMS1P1 | -5.3802907 | 4.78E-61 |
| PIK3CD-AS1 | 3.939302 | 5.76E-57 |
| CELF2-AS1 | 3.8846333 | 1.77E-55 |
| LINC00174 | 4.8940334 | 2.23E-54 |
| C1RL-AS1 | 3.0544217 | 5.99E-54 |
| ATP11A-AS1 | 5.6292454 | 2.56E-53 |
| LINC00861 | -4.7902916 | 2.56E-52 |
| FAM157A | 4.8071763 | 2.96E-52 |
| VPS9D1-AS1 | 3.3272544 | 1.85E-46 |
| SNHG14 | -3.3819482 | 2.01E-46 |
| FAM157C | 4.4078013 | 5.35E-46 |
| TREML3P | 5.353592 | 1.48E-44 |
| BACH1-IT2 | 4.5914613 | 3.72E-42 |
| MRTFA-AS1 | 4.0161541 | 1.12E-39 |
| PRKCQ-AS1 | -4.3063015 | 5.54E-39 |
| SVIL-AS1 | 2.7361288 | 7.95E-39 |
| LINC01503 | 4.2638659 | 8.32E-39 |
| BASP1-AS1 | 5.1608956 | 1.69E-38 |
| SLC12A5-AS1 | 5.0985193 | 3.39E-38 |
| PRR7-AS1 | 2.9266074 | 6.75E-38 |
| LINC01347 | 4.3433101 | 1.14E-37 |
| PIK3CD-AS2 | 4.3546278 | 1.53E-37 |
| GAS5 | -2.9550688 | 2.3E-37 |
| LINC00862 | 5.3531576 | 2.59E-37 |
| ARHGAP15-AS1 | 3.2731076 | 6.66E-36 |
| TET2-AS1 | 4.2874287 | 1.11E-35 |
| DLEU1 | 2.9343301 | 5.61E-35 |
| ARHGAP26-IT1 | 4.1393903 | 6.25E-35 |
| ANKRD44-IT1 | 3.1880641 | 8.03E-35 |
| ARAP1-AS2 | 2.9938932 | 2.82E-34 |
| DPYD-AS2 | 3.7706301 | 8.02E-34 |
| GTF2IRD1P1 | 2.8569148 | 1.53E-33 |
| PXN-AS1 | 2.3598402 | 5.93E-32 |
| LINC02035 | 3.9222437 | 6.87E-32 |
| SLC8A1-AS1 | 4.5996716 | 6.95E-32 |
| ECE1-AS1 | 4.5370171 | 1.36E-31 |
| ZSWIM8-AS1 | 1.7820602 | 2.61E-31 |
| ENTPD1-AS1 | 3.4449238 | 3.85E-31 |
| ADAMTSL4-AS2 | 3.4382273 | 1.3E-30 |
| ASAP1-IT2 | 5.688625 | 1.78E-30 |
| IRAIN | 2.6396625 | 2.2E-30 |
| LINC00513 | 4.0086314 | 9.52E-30 |
| SNHG1 | -3.0051546 | 1.26E-29 |
| ABALON | -5.2805953 | 2.12E-29 |
| ARHGAP26-AS1 | 3.6116547 | 4.25E-29 |
| LINC02751 | 4.281043 | 9.96E-29 |
| CSNK1G2-AS1 | 5.8533539 | 1.11E-28 |
| KCNQ1OT1 | 2.7255327 | 3.72E-28 |
| LINC00937 | 3.6484775 | 5.49E-28 |
| IL10RB-DT | 2.8181058 | 5.99E-28 |
| LINC02362 | 3.6402272 | 9.5E-28 |
| SND1-IT1 | 3.4489814 | 1.13E-27 |
| LINC00402 | -5.4764823 | 1.32E-27 |
| ARHGAP27P1-BPTFP1-KPNA2P3 | 2.0735424 | 1.01E-26 |
| MKNK1-AS1 | 3.9676418 | 2.52E-26 |
| LINC02363 | 4.5306972 | 2.58E-26 |
| KMT2E-AS1 | 2.057142 | 2.89E-26 |
| CYP1B1-AS1 | 7.3621765 | 4.33E-26 |
| TRG-AS1 | -4.1508632 | 5.37E-26 |
| LIPC-AS1 | 5.7677324 | 8.92E-26 |
| AIRN | 3.9672693 | 1.13E-25 |
| USP3-AS1 | 2.4048518 | 1.25E-25 |
| TERC | -3.0496624 | 4.58E-25 |
| CFLAR-AS1 | 2.8356076 | 9.8E-25 |
| SNHG8 | -3.2342392 | 1.23E-24 |
| PCED1B-AS1 | -3.300456 | 1.72E-24 |
| PPP4R1L | 2.6272287 | 1.92E-24 |
| DISC1-IT1 | 4.564535 | 3.84E-24 |
| LINC02067 | -3.8394871 | 3.95E-24 |
| LINC00963 | 2.2062873 | 7.09E-24 |
| LINC01002 | 4.5494559 | 8.73E-24 |
| CLUHP3 | -4.5431288 | 8.82E-24 |
| CYP2T1P | 3.1268419 | 1.06E-23 |
| SBF2-AS1 | 2.6988962 | 1.25E-23 |
| IDI2-AS1 | 2.7934605 | 2.79E-23 |
| HORMAD2-AS1 | 2.2001418 | 3.13E-23 |
| LGALSL-DT | 4.7094454 | 4.78E-23 |
| GSEC | 2.6431973 | 5.91E-23 |
| LINC01619 | 2.2611749 | 7.65E-23 |
| DGCR11 | 3.1460057 | 1.13E-22 |
| SNHG32 | -2.7461812 | 1.36E-22 |
| CEBPB-AS1 | 2.1778641 | 2.86E-22 |
| LINC00173 | 3.2020903 | 5.15E-22 |
| DAAM2-AS1 | 6.2618087 | 6.78E-22 |
| ITPK1-AS1 | 3.1994483 | 1.07E-21 |
| RARA-AS1 | 2.0761169 | 1.21E-21 |
| UBOX5-AS1 | 2.8784516 | 1.31E-21 |
| N4BP2L2-IT2 | 2.5656174 | 1.68E-21 |
| NBPF25P | 2.6507608 | 3.56E-21 |
| CLIP1-AS1 | 5.0718135 | 4.53E-21 |
| PLEKHM1P1 | 1.6969328 | 5.09E-21 |
| TBC1D22A-AS1 | 4.1855114 | 6.19E-21 |
| LINC02207 | 4.8253896 | 6.26E-21 |
| DOCK8-AS1 | 2.3353641 | 6.59E-21 |
| LINC00989 | -4.3229865 | 1.64E-20 |
| HIF1A-AS3 | 4.9054593 | 1.69E-20 |
| MAP3K5-AS1 | 3.5195711 | 2.02E-20 |
| LINC01550 | -5.0983423 | 2.14E-20 |
| HIF1A-AS1 | 2.5659672 | 3.7E-20 |
| LINC00649 | -4.3741683 | 4.31E-20 |
| ABCC13 | -11.420217 | 6.3E-20 |
| NPTN-IT1 | 3.0225805 | 8.37E-20 |
| CXCR2P1 | -3.9322913 | 8.97E-20 |
| SBDSP1 | -2.7232859 | 1.17E-19 |
| CATIP-AS1 | 3.0124465 | 1.34E-19 |
| SYNE1-AS1 | 3.1230772 | 1.34E-19 |
| ADAMTSL4-AS1 | 3.3205298 | 2.27E-19 |
| MMP25-AS1 | 3.0878219 | 2.56E-19 |
| DTX2P1-UPK3BP1-PMS2P11 | 2.2609309 | 3.45E-19 |
| LEF1-AS1 | -4.9022561 | 3.56E-19 |
| DANCR | -3.83624 | 3.93E-19 |
| NSUN5P2 | -2.1529068 | 1.51E-18 |
| BMS1P14 | 6.7815208 | 1.66E-18 |
| ADORA2A-AS1 | 4.3743216 | 1.68E-18 |
| LINC02210 | -3.8798985 | 2.12E-18 |
| FAM13A-AS1 | 2.0597898 | 2.12E-18 |
| LINC01004 | 2.2115898 | 4.59E-18 |
| MAPKAPK5-AS1 | -1.9378238 | 4.98E-18 |
| LINC02446 | -5.5521651 | 5.04E-18 |
| MIR3936HG | 2.2007649 | 7.32E-18 |
| LINC02019 | 3.721999 | 9.98E-18 |
| LINC00676 | 5.4890813 | 1.12E-17 |
| HERC2P2 | -2.7037504 | 1.45E-17 |
| LINC01988 | 3.9042351 | 1.9E-17 |
| LINC02772 | -10.579419 | 2.02E-17 |
| LINC02273 | -5.9230154 | 2.57E-17 |
| ACSL3-AS1 | 4.0520764 | 2.74E-17 |
| FAM238A | 3.2357269 | 3.7E-17 |
| ELMO1-AS1 | 2.1234503 | 6.91E-17 |
| ANKRD36BP2 | -8.4715959 | 1.06E-16 |
| LINC01993 | 5.3332033 | 1.87E-16 |
| FOXP1-AS1 | 3.1099062 | 1.96E-16 |
| PTENP1 | 1.8757178 | 2.49E-16 |
| SNRK-AS1 | 1.3206196 | 3.82E-16 |
| WDFY3-AS2 | 4.3017791 | 4.26E-16 |
| EPB41L4A-AS1 | -3.2807388 | 5.83E-16 |
| CD99P1 | 2.6177297 | 8.71E-16 |
| LINC02604 | 4.439201 | 1.06E-15 |
| JARID2-AS1 | 2.9402963 | 1.51E-15 |
| MIF-AS1 | -1.8640144 | 1.57E-15 |
| TMEM14EP | 2.1677342 | 2.74E-15 |
| ZNF542P | -4.0939239 | 3.23E-15 |
| TP73-AS1 | -2.3820118 | 3.53E-15 |
| C2CD4D-AS1 | -5.20165 | 6.24E-15 |
| ITPRIP-AS1 | 3.6653318 | 6.76E-15 |
| STARD7-AS1 | 2.257699 | 8.91E-15 |
| DHRS4-AS1 | -2.7694911 | 9.96E-15 |
| SH3BP5-AS1 | 1.4692383 | 1.13E-14 |
| GTF2IP13 | 3.3833294 | 1.58E-14 |
| INE1 | 2.6525509 | 1.78E-14 |
| LINC01303 | 2.3785387 | 1.84E-14 |
| SMPD5 | 8.3077322 | 2.07E-14 |
| MIR3945HG | 3.2174769 | 2.08E-14 |
| PHKA2-AS1 | 2.0884188 | 2.51E-14 |
| PVT1 | -2.6566723 | 2.65E-14 |
| OSER1-DT | -2.0071521 | 2.81E-14 |
| FRG1CP | -3.4345302 | 4.24E-14 |
| LINC-PINT | 2.4599926 | 4.31E-14 |
| DGCR5 | 5.5037365 | 4.4E-14 |
| TMEM147-AS1 | -2.8674023 | 6.07E-14 |
| CD27-AS1 | -1.9569421 | 6.51E-14 |
| LINC00102 | 4.0861062 | 8.02E-14 |
| SEMA3F-AS1 | 1.2731034 | 1.07E-13 |
| DPY19L2P2 | -4.5322306 | 1.23E-13 |
| LUCAT1 | 1.7199198 | 1.25E-13 |
| ATP2C2-AS1 | 3.7268645 | 1.64E-13 |
| NPEPPSP1 | 1.3539058 | 1.69E-13 |
| LINC01948 | 4.2001247 | 1.94E-13 |
| LINC00570 | -9.4826477 | 1.95E-13 |
| DDX11L10 | -3.5835518 | 2.32E-13 |
| IPO5P1 | -2.2634188 | 2.62E-13 |
| RNF157-AS1 | -3.630441 | 3.26E-13 |
| LINC01531 | 3.8304315 | 3.36E-13 |
| TPTE2P5 | -2.8373567 | 3.4E-13 |
| DIAPH1-AS1 | 2.710318 | 4.58E-13 |
| ATP6V0E2-AS1 | -5.4157794 | 5.76E-13 |
| LPP-AS2 | 3.2936653 | 6.09E-13 |
| SNHG5 | -2.7649688 | 6.59E-13 |
| MIR194-2HG | 4.8106158 | 6.67E-13 |
| RPS6KA2-AS1 | 4.2034737 | 8.54E-13 |
| MIR22HG | 3.0527441 | 8.95E-13 |
| ADNP-AS1 | 2.7978207 | 9.04E-13 |
| PSMD6-AS2 | 2.2754867 | 9.65E-13 |
| GVINP1 | -3.1978459 | 1.31E-12 |
| LINC02288 | 3.7104222 | 1.48E-12 |
| ST20-AS1 | 1.6255062 | 1.48E-12 |
| LINC00667 | -2.132747 | 1.66E-12 |
| SAP30L-AS1 | 1.8248534 | 2.26E-12 |
| LINC01547 | 1.9738521 | 2.4E-12 |
| RRN3P1 | -2.5683514 | 2.77E-12 |
| ADPGK-AS1 | 1.6063629 | 3.06E-12 |
| TRPM2-AS | 5.1674492 | 3.78E-12 |
| GTF2IP12 | 3.1657592 | 3.81E-12 |
| RNF216-IT1 | 3.9737945 | 3.87E-12 |
| TPI1P2 | 3.8221411 | 4.71E-12 |
| MRPS31P5 | -2.3256782 | 5.69E-12 |
| UCKL1-AS1 | 2.3805921 | 5.7E-12 |
| LINC02361 | -3.3659162 | 5.77E-12 |
| SLC5A4-AS1 | -4.6229535 | 7.16E-12 |
| LINC01093 | 4.8106509 | 7.45E-12 |
| PEG13 | 1.9623069 | 7.86E-12 |
| HCG18 | -1.7791131 | 7.94E-12 |
| LINC01629 | 5.7286863 | 8.23E-12 |
| NINJ2-AS1 | 1.8475997 | 8.86E-12 |
| SATB1-AS1 | -5.1065719 | 9.48E-12 |
| GTF2IP4 | 1.5645725 | 1.04E-11 |
| LINC02803 | 3.7638022 | 1.12E-11 |
| LINC00612 | -3.1158725 | 1.18E-11 |
| CELSR1P1 | 3.26396 | 1.18E-11 |
| GOLGA2P5 | -2.4890528 | 1.19E-11 |
| MAP3K20-AS1 | 3.0240481 | 1.23E-11 |
| PRSS30P | -6.0543316 | 1.33E-11 |
| GSN-AS1 | 2.1221602 | 1.46E-11 |
| ZRANB2-AS1 | -2.3090699 | 1.47E-11 |
| FTX | 2.0071209 | 1.55E-11 |
| DNAH17-AS1 | 8.1789741 | 1.81E-11 |
| LINC00987 | -2.7543168 | 1.84E-11 |
| BCL2L1-AS1 | 2.1847584 | 1.88E-11 |
| WHAMMP3 | -2.6978978 | 2.38E-11 |
| LINC00528 | 3.0896406 | 3.4E-11 |
| LINC02680 | 2.412582 | 3.49E-11 |
| LINC00398 | 3.5332768 | 3.72E-11 |
| PLBD1-AS1 | 2.3253114 | 3.72E-11 |
| EPS15-AS1 | 2.733429 | 4.36E-11 |
| ITIH4-AS1 | 2.7635861 | 4.67E-11 |
| RBM5-AS1 | 1.3331621 | 5.57E-11 |
| DNAAF4-CCPG1 | 1.8374765 | 5.89E-11 |
| LINC00671 | 2.8033537 | 7.12E-11 |
| GOLGA2P10 | -5.2862932 | 7.23E-11 |
| BMS1P2 | -2.4282767 | 8.75E-11 |
| KCNJ2-AS1 | 3.4475102 | 9.13E-11 |
| BEAN1-AS1 | 4.0590841 | 9.85E-11 |
| SNHG10 | 1.1209185 | 9.98E-11 |
| DDN-AS1 | 1.7871546 | 1.02E-10 |
| RNF213-AS1 | 3.1770165 | 1.07E-10 |
| SP2-AS1 | 2.7117456 | 1.18E-10 |
| NAPSB | -2.2004465 | 1.24E-10 |
| GUSBP11 | 1.4466976 | 1.25E-10 |
| LINC01871 | -4.6134842 | 1.31E-10 |
| ZFAS1 | 1.4624178 | 1.42E-10 |
| HERC2P3 | -2.0853239 | 1.42E-10 |
| SMG1P7 | -2.9676305 | 1.68E-10 |
| FAM111A-DT | -2.9765612 | 1.85E-10 |
| ARMCX5-GPRASP2 | -2.3011746 | 1.9E-10 |
| WHAMMP2 | -3.3634292 | 2.66E-10 |
| SH3GL1P1 | 2.2536705 | 2.71E-10 |
| DPYD-AS1 | 2.687384 | 2.76E-10 |
| RPL23AP53 | -3.5151377 | 2.77E-10 |
| TRBV11-2 | -4.6644403 | 2.9E-10 |
| WARS2-AS1 | -3.2566529 | 3.07E-10 |
| MIR181A1HG | 2.9475389 | 3.57E-10 |
| ZNF337-AS1 | -2.7386961 | 3.71E-10 |
| CPEB1-AS1 | 7.7297033 | 3.87E-10 |
| LINC00641 | 2.3041567 | 4.28E-10 |
| DSCAS | 3.1188835 | 4.55E-10 |
| MIR3142HG | -5.0354135 | 4.89E-10 |
| NSUN5P1 | -1.7235023 | 5.58E-10 |
| SERHL | -2.4527552 | 6.98E-10 |
| PCBP1-AS1 | 1.4466292 | 6.98E-10 |
| NNT-AS1 | -3.3232288 | 7E-10 |
| LINC02648 | -4.4085295 | 7.91E-10 |
| LINC02456 | -7.2131205 | 7.95E-10 |
| TTN-AS1 | 1.4912356 | 8.19E-10 |
| TMLHE-AS1 | 2.7219974 | 8.87E-10 |
| LINC00921 | 1.7687444 | 9E-10 |
| MAFA-AS1 | 7.2439446 | 9.12E-10 |
| LINC00680 | -3.6887437 | 9.45E-10 |
| LPGAT1-AS1 | 4.3216049 | 9.52E-10 |
| LINC01943 | 2.2765902 | 9.54E-10 |
| MIR9-3HG | -4.2192392 | 9.59E-10 |
| COL4A2-AS1 | 7.3004155 | 9.95E-10 |
| HERC2P9 | -1.9876269 | 1.02E-09 |
| FAF1-AS1 | 2.6522713 | 1.03E-09 |
| LINC01259 | -7.6947115 | 1.18E-09 |
| PPT2-EGFL8 | -2.0926386 | 1.24E-09 |
| LINC01278 | -2.6359748 | 1.27E-09 |
| LINC02084 | -6.6839214 | 1.27E-09 |
| KLRA1P | -4.7292977 | 1.34E-09 |
| C1QTNF7-AS1 | 2.4255398 | 1.42E-09 |
| RASA4CP | -2.3432789 | 1.49E-09 |
| ELF3-AS1 | 3.1283532 | 1.76E-09 |
| LINC02320 | 4.3357696 | 1.76E-09 |
| LINC02212 | 4.0135867 | 1.79E-09 |
| ZNF350-AS1 | 2.8161953 | 1.82E-09 |
| LINC02520 | -4.6631671 | 1.84E-09 |
| SOCAR | 3.0828796 | 1.88E-09 |
| ANAPC1P1 | -5.1438026 | 1.9E-09 |
| CKMT2-AS1 | -3.141953 | 1.93E-09 |
| TBC1D8-AS1 | 5.3057371 | 1.93E-09 |
| ADCY10P1 | 1.9583385 | 2.21E-09 |
| ADD3-AS1 | 1.7721373 | 2.38E-09 |
| TMPO-AS1 | -2.5884335 | 3.18E-09 |
| TMEM191A | -2.7947639 | 3.41E-09 |
| HLA-DRB6 | -4.0587242 | 3.47E-09 |
| FAM53B-AS1 | 3.1633865 | 3.64E-09 |
| GTF2IP1 | 1.516931 | 3.84E-09 |
| DOCK4-AS1 | 3.5659565 | 4.01E-09 |
| LINC01191 | 2.5521044 | 4.03E-09 |
| LINC01160 | 3.8387379 | 4.34E-09 |
| NDUFV2-AS1 | 1.9027803 | 4.66E-09 |
| EIF1B-AS1 | 2.4978822 | 4.73E-09 |
| PLEKHA8P1 | -3.1346086 | 5.03E-09 |
| GHRLOS | 1.6864115 | 5.22E-09 |
| LINC01772 | 1.7418698 | 5.46E-09 |
| KRT73-AS1 | -5.5457692 | 6.18E-09 |
| DNMBP-AS1 | 2.5523791 | 6.35E-09 |
| AOAH-IT1 | 2.6512638 | 6.57E-09 |
| ZNF286B | -4.7112342 | 7E-09 |
| HSD11B1-AS1 | 3.6121037 | 9.55E-09 |
| GUSBP1 | -2.3367562 | 9.82E-09 |
| LPAL2 | -5.3514933 | 1.07E-08 |
| SLCO4A1-AS1 | 5.3783181 | 1.08E-08 |
| MROCKI | 4.4139007 | 1.13E-08 |
| UBBP4 | -5.267168 | 1.13E-08 |
| SENCR | 1.7039225 | 1.2E-08 |
| SNCA-AS1 | -7.3791682 | 1.32E-08 |
| GBAP1 | 1.4031221 | 1.39E-08 |
| SUGT1P4-STRA6LP | -7.4050677 | 1.42E-08 |
| SLC25A5-AS1 | -1.3565646 | 1.53E-08 |
| LINC02193 | 3.4173312 | 1.61E-08 |
| ULK4P2 | 3.1993192 | 1.97E-08 |
| LINC02615 | -4.7985886 | 2.1E-08 |
| LINC02833 | 7.3713435 | 2.2E-08 |
| NRADDP | 2.2455305 | 2.45E-08 |
| NCF4-AS1 | 1.4995331 | 2.61E-08 |
| IATPR | -7.0398742 | 2.93E-08 |
| LINC01579 | 6.8211015 | 3.14E-08 |
| LINC01973 | 2.8529253 | 3.21E-08 |
| CD2BP2-DT | 1.9210511 | 3.53E-08 |
| APOA1-AS | 3.3757844 | 3.55E-08 |
| PAN3-AS1 | 1.2583928 | 3.61E-08 |
| JAZF1-AS1 | -2.9514934 | 3.67E-08 |
| KIAA1614-AS1 | 3.3399453 | 3.68E-08 |
| APTR | 1.4909913 | 3.71E-08 |
| ZEB2-AS1 | 1.8056509 | 3.83E-08 |
| NBPF8 | 1.6275954 | 4.17E-08 |
| IL6R-AS1 | 2.5822601 | 4.39E-08 |
| RUNDC3A-AS1 | -7.020543 | 4.46E-08 |
| LINC02384 | -3.1073365 | 4.58E-08 |
| LINC00663 | -3.679641 | 4.64E-08 |
| LINC01127 | 2.6566049 | 5.11E-08 |
| MAP3K2-DT | 1.7288745 | 5.31E-08 |
| OR6W1P | 6.9508458 | 5.36E-08 |
| LINC01934 | -3.2927217 | 5.53E-08 |
| SDHAP3 | -2.267887 | 5.8E-08 |
| SNHG12 | -1.3456539 | 5.88E-08 |
| SDCBP2-AS1 | 2.2174154 | 6.51E-08 |
| UBXN7-AS1 | 2.0759404 | 7.28E-08 |
| LINC01011 | -2.2642524 | 7.57E-08 |
| NUTM2B-AS1 | 2.1724837 | 7.9E-08 |
| LINC02295 | -7.1119427 | 7.96E-08 |
| MIR29B2CHG | 2.0445954 | 8.24E-08 |
| IER3-AS1 | 3.8873946 | 8.34E-08 |
| KDM7A-DT | -2.6258971 | 8.77E-08 |
| SNHG29 | -2.2098852 | 8.83E-08 |
| TLR8-AS1 | 2.8747839 | 8.92E-08 |
| MIPEPP3 | 1.6468364 | 8.97E-08 |
| LINC02284 | 2.3830552 | 9.36E-08 |
| MIAT | 1.6235269 | 9.66E-08 |
| BTBD9-AS1 | 2.708457 | 9.7E-08 |
| SEMA3B-AS1 | 3.4772034 | 1.01E-07 |
| RPL23AP87 | 3.1938246 | 1.08E-07 |
| ILF3-DT | -1.7087378 | 1.14E-07 |
| SNHG4 | -3.484436 | 1.34E-07 |
| BIRC6-AS2 | 3.2131334 | 1.35E-07 |
| RAP2C-AS1 | 2.5504542 | 1.37E-07 |
| IQCH-AS1 | -2.8892254 | 1.41E-07 |
| LINC01482 | 6.6030802 | 1.47E-07 |
| DOCK9-DT | -4.5275093 | 0.00000016 |
| KCNMA1-AS3 | 9.4442444 | 1.66E-07 |
| UVRAG-DT | 3.4485568 | 1.86E-07 |
| LINC00885 | 4.081281 | 2.03E-07 |
| C2orf27A | -5.7615751 | 0.00000021 |
| LINC02812 | -4.909325 | 2.18E-07 |
| H2BP2 | 2.3736949 | 2.23E-07 |
| LINC01806 | -2.612372 | 2.28E-07 |
| LINC02287 | 4.5401664 | 2.35E-07 |
| LOXL1-AS1 | 1.6548539 | 2.51E-07 |
| LINC00623 | -1.3555076 | 2.52E-07 |
| EMBP1 | 1.3239704 | 2.67E-07 |
| LINC00426 | -2.9131212 | 2.82E-07 |
| DPP9-AS1 | 1.244247 | 2.85E-07 |
| TSPY26P | 3.1026042 | 3.21E-07 |
| PABPC4-AS1 | -1.6701685 | 3.29E-07 |
| DIAPH2-AS1 | 4.0822162 | 3.37E-07 |
| LINC00399 | 4.5499438 | 3.53E-07 |
| DIP2A-IT1 | 3.0518542 | 3.66E-07 |
| LINC02705 | 2.7198645 | 3.76E-07 |
| TSC22D1-AS1 | -2.7586006 | 3.78E-07 |
| LINC02325 | -3.7130325 | 4.06E-07 |
| GABPB1-IT1 | 1.6765043 | 4.43E-07 |
| TGFA-IT1 | 4.6745789 | 4.57E-07 |
| LINC00869 | -1.5428278 | 0.00000048 |
| LINC01128 | -2.235232 | 4.85E-07 |
| BACH1-IT3 | 2.9482625 | 4.86E-07 |
| TUG1 | 0.9605818 | 5.08E-07 |
| MMADHC-DT | 1.1576352 | 5.29E-07 |
| C9orf163 | 2.4524995 | 0.00000053 |
| CACNA1C-AS1 | -5.5646117 | 0.00000053 |
| CROCCP2 | -2.1058758 | 5.32E-07 |
| HOXB-AS1 | -3.080447 | 5.55E-07 |
| WDR86-AS1 | -3.8770128 | 5.77E-07 |
| SNHG15 | -1.8635945 | 6.09E-07 |
| NCF1B | 1.6153456 | 6.28E-07 |
| LCMT1-AS2 | 2.7801242 | 6.32E-07 |
| LINC02328 | -2.7891543 | 6.48E-07 |
| THAP7-AS1 | -3.1295236 | 0.00000067 |
| SNHG6 | -1.6265349 | 6.72E-07 |
| ZNF815P | -1.3745363 | 6.93E-07 |
| OR2A20P | -3.336686 | 7.17E-07 |
| LINC00640 | 6.0582274 | 7.18E-07 |
| SNHG22 | -2.9038411 | 7.92E-07 |
| LINC01355 | -3.0177558 | 8.15E-07 |
| UBE2Q2P1 | 2.4727099 | 8.29E-07 |
| FAM86DP | -2.4863071 | 8.29E-07 |
| LINC01311 | -4.124101 | 0.00000086 |
| ZNF876P | -3.7900963 | 0.00000091 |
| ZNF32-AS1 | -4.1748034 | 9.19E-07 |
| CD81-AS1 | -2.1156537 | 0.00000103 |
| LINC01977 | 5.6464091 | 0.00000126 |
| HMGA2-AS1 | 3.2854598 | 0.00000175 |
| HAR1A | -5.5800042 | 0.00000183 |
| DENND6A-DT | 4.6206272 | 0.00000186 |
| FAM87B | 2.0970719 | 0.00000192 |
| LINC00926 | -2.3769742 | 0.00000192 |
| CLRN1-AS1 | 5.9459747 | 0.00000201 |
| LINC00996 | -6.3883343 | 0.00000205 |
| LINC01572 | -2.2912596 | 0.00000218 |
| CCDC144CP | -2.3515505 | 0.00000238 |
| NDUFB2-AS1 | -1.9526299 | 0.0000027 |
| LINC00342 | -1.6069958 | 0.00000283 |
| ZNF687-AS1 | 3.1294838 | 0.00000331 |
| ARHGEF35-AS1 | -2.361918 | 0.00000332 |
| OR2A1-AS1 | -2.6906833 | 0.00000361 |
| SCAANT1 | 2.5694763 | 0.00000368 |
| LINC01703 | 5.4077319 | 0.00000369 |
| MDS2 | -5.129189 | 0.00000374 |
| ARHGAP31-AS1 | 4.5690344 | 0.00000377 |
| DACT3-AS1 | 3.9628815 | 0.0000038 |
| LINC01730 | -4.0460063 | 0.0000038 |
| USP12-AS1 | 3.5720302 | 0.00000419 |
| LINC01801 | -4.41505 | 0.00000425 |
| LINC01891 | -5.851893 | 0.00000428 |
| VCAN-AS1 | 2.962448 | 0.00000429 |
| TTC28-AS1 | -1.3199479 | 0.00000439 |
| LINC02213 | 3.8139516 | 0.00000441 |
| TPTEP2 | 2.1467639 | 0.00000479 |
| OR2A9P | -2.7804295 | 0.00000486 |
| DTNB-AS1 | 2.6115394 | 0.00000487 |
| ZNF32-AS2 | -4.4015008 | 0.00000493 |
| GOLGA2P7 | -4.7359929 | 0.00000502 |
| ZNF667-AS1 | -3.8855363 | 0.00000506 |
| HLX-AS1 | 1.5436792 | 0.00000561 |
| LINC02316 | -1.540741 | 0.0000058 |
| BICDL3P | 3.1806324 | 0.00000598 |
| PTOV1-AS2 | 0.8626469 | 0.00000609 |
| LINC01991 | 3.7570198 | 0.00000613 |
| RMST | -6.3815169 | 0.00000619 |
| OIP5-AS1 | -1.281963 | 0.00000704 |
| LINC01816 | 2.6854107 | 0.00000706 |
| LINC02034 | 5.040991 | 0.00000707 |
| ZNF767P | -1.1090481 | 0.00000727 |
| MIR133A1HG | 2.0701552 | 0.00000773 |
| CA3-AS1 | -3.154572 | 0.0000079 |
| DBH-AS1 | -5.7301014 | 0.00000814 |
| CACTIN-AS1 | -1.418599 | 0.00000833 |
| ALOX15P1 | -4.1318198 | 0.00000857 |
| NCMAP-DT | -2.9830203 | 0.00000859 |
| TP53TG1 | -1.9416135 | 0.00000872 |
| LINC02018 | 2.6923285 | 0.00000938 |
| STXBP5-AS1 | 1.1803097 | 0.00001 |
| RAD51-AS1 | -1.2939899 | 0.0000102 |
| GUSBP2 | -1.6106844 | 0.0000104 |
| LIMD1-AS1 | -1.5153009 | 0.0000105 |
| LINC00654 | 2.1432745 | 0.0000107 |
| LINC02603 | 1.5927664 | 0.0000107 |
| WAC-AS1 | 1.2510005 | 0.0000108 |
| FAM27E3 | 2.0659229 | 0.0000108 |
| CHMP1B2P | -5.7113332 | 0.0000114 |
| LINC00865 | -5.9915525 | 0.0000116 |
| ASH1L-AS1 | 1.247617 | 0.0000118 |
| OBI1-AS1 | -1.710656 | 0.0000121 |
| ZNF213-AS1 | -1.7261468 | 0.0000123 |
| POC1B-AS1 | 1.4524372 | 0.0000135 |
| LGALS8-AS1 | 1.4910775 | 0.0000136 |
| CLMAT3 | -2.5106058 | 0.0000138 |
| GTF2IP20 | 1.5474028 | 0.000014 |
| CARNMT1-AS1 | -2.1090735 | 0.0000141 |
| FAM226B | -5.68874 | 0.0000146 |
| LINC02785 | 3.1340142 | 0.0000152 |
| LINC00299 | -2.3140351 | 0.0000157 |
| FGD5-AS1 | 0.7844683 | 0.0000161 |
| CACNA1C-AS2 | -5.8870585 | 0.0000162 |
| MIR4435-2HG | 1.1612666 | 0.0000179 |
| LINC00115 | 1.4795444 | 0.0000183 |
| MAST4-AS1 | 3.1247795 | 0.0000184 |
| UXT-AS1 | -1.4872719 | 0.0000185 |
| LINC00630 | -1.979926 | 0.0000193 |
| LINC01624 | -3.0218535 | 0.0000195 |
| ABCA11P | -1.3167605 | 0.0000203 |
| TPM1-AS | -2.7363588 | 0.0000205 |
| C21orf62-AS1 | -3.8345615 | 0.0000209 |
| LINC00852 | 1.5251462 | 0.0000212 |
| PRR34-AS1 | 2.0767023 | 0.0000214 |
| RPL23AP82 | -1.7564057 | 0.0000216 |
| GNG12-AS1 | 1.8685961 | 0.0000233 |
| CHRM3-AS2 | -3.2017661 | 0.000025 |
| LINC00944 | -4.0803482 | 0.0000273 |
| SMILR | -5.6107343 | 0.0000281 |
| GAS5-AS1 | -1.6350611 | 0.0000283 |
| RNF216P1 | -1.4858254 | 0.0000288 |
| LINC02289 | 2.4239609 | 0.0000299 |
| LINC02100 | -3.5012044 | 0.000032 |
| LYPLAL1-DT | -5.5828586 | 0.0000323 |
| LINC01176 | 1.7143527 | 0.0000328 |
| MIR762HG | -1.3650984 | 0.0000332 |
| FAHD2CP | -4.1552033 | 0.0000337 |
| ZNF890P | -2.6429939 | 0.0000358 |
| RCAN3AS | -4.7307982 | 0.0000363 |
| LINC00968 | 2.1538013 | 0.0000371 |
| LINC00526 | -5.3972347 | 0.0000371 |
| DICER1-AS1 | 1.5421207 | 0.0000378 |
| IDH2-DT | -1.9471631 | 0.0000405 |
| STARD4-AS1 | -2.0919287 | 0.0000427 |
| PSMA3-AS1 | 0.8423923 | 0.0000436 |
| ANKRD36BP1 | 1.5136321 | 0.0000444 |
| LINC00853 | -5.3802147 | 0.0000461 |
| LINC01260 | -4.7791727 | 0.0000463 |
| ZNF433-AS1 | 1.1258794 | 0.0000465 |
| FAAHP1 | -3.2497726 | 0.0000469 |
| SCARNA9 | 1.3047365 | 0.0000472 |
| LINC00189 | 2.2405963 | 0.0000482 |
| NUTM2A-AS1 | -1.570017 | 0.0000485 |
| POLR2J4 | 0.952585 | 0.0000513 |
| FUT8-AS1 | -3.1927265 | 0.0000515 |
| ZNF436-AS1 | -2.5906133 | 0.0000516 |
| LINC02611 | -2.902163 | 0.0000532 |
| FAM153CP | -4.6386523 | 0.0000557 |
| SNAI3-AS1 | 1.368829 | 0.0000573 |
| FARSA-AS1 | -2.3354813 | 0.0000592 |
| SIRPG-AS1 | -2.9143227 | 0.0000652 |
| USP32P3 | -1.7390448 | 0.0000659 |
| SCAMP1-AS1 | -2.2689746 | 0.000066 |
| SCAT1 | 2.2443991 | 0.0000724 |
| FBXL19-AS1 | 1.4105926 | 0.0000732 |
| KRT17P2 | -5.459096 | 0.0000749 |
| PSORS1C3 | 2.2531301 | 0.0000783 |
| BMS1P4 | -1.4155479 | 0.000082 |
| APOBEC3B-AS1 | 1.201243 | 0.0000823 |
| DNAJC3-DT | 1.4520867 | 0.0000863 |
| LINC01684 | -3.382586 | 0.000102 |
| EXOSC10-AS1 | -2.0736502 | 0.000103 |
| LINC01410 | 1.136005 | 0.000103 |
| FAM86EP | -5.0995004 | 0.000103 |
| LINC02478 | 2.87137 | 0.000104 |
| STX18-AS1 | -2.8314668 | 0.000105 |
| ULK4P3 | 2.9464828 | 0.000107 |
| ESRG | -6.1130565 | 0.000111 |
| DLGAP1-AS2 | 1.5307653 | 0.000113 |
| RDM1P5 | -5.1215237 | 0.000119 |
| SSR4P1 | -3.5599772 | 0.000121 |
| ZBTB20-AS1 | 1.5930189 | 0.000124 |
| LINC00920 | -4.4165442 | 0.000124 |
| TIMM23B-AGAP6 | -1.2500489 | 0.00013 |
| LINC00892 | -3.0660821 | 0.00013 |
| LINC01473 | -5.0555176 | 0.000133 |
| ZNF528-AS1 | -2.8678437 | 0.000136 |
| CT70 | 2.9200313 | 0.000137 |
| C1orf220 | -3.2341157 | 0.00014 |
| LINC02700 | 3.3995857 | 0.000146 |
| FAM86C2P | -5.1169783 | 0.000147 |
| LINC02397 | -3.2310201 | 0.00015 |
| ZMYM4-AS1 | -1.7520331 | 0.00015 |
| NCAM1-AS1 | -5.147396 | 0.000152 |
| NRAV | -3.5835018 | 0.000153 |
| LINC02693 | -2.1945099 | 0.000154 |
| GRPEL2-AS1 | -3.1158539 | 0.000155 |
| HLA-DQB1-AS1 | -3.0749991 | 0.000162 |
| B4GALT4-AS1 | 1.6294719 | 0.000171 |
| ALOX12P2 | 1.6694934 | 0.000171 |
| HM13-AS1 | 1.7409829 | 0.000173 |
| HMGN3-AS1 | -1.7399351 | 0.000177 |
| RPS18P9 | 1.8261457 | 0.000181 |
| VASH1-AS1 | -1.7231384 | 0.000182 |
| LINC00888 | -2.9195739 | 0.000187 |
| TCL6 | -4.0503214 | 0.000196 |
| LINC01890 | 1.2553898 | 0.000196 |
| MSL3P1 | -3.9046326 | 0.000202 |
| SERPINB9P1 | 2.0749476 | 0.000208 |
| HECW2-AS1 | -2.561114 | 0.000213 |
| LINC00205 | -1.2457692 | 0.000218 |
| ROCK1P1 | 0.9662226 | 0.000223 |
| MORC2-AS1 | -1.6782609 | 0.000226 |
| PRNCR1 | 1.1791222 | 0.000226 |
| LINC00539 | 1.6469985 | 0.000231 |
| MIR646HG | 1.3643649 | 0.000245 |
| OBSCN-AS1 | -2.3774682 | 0.000247 |
| GNRHR2 | -1.0510137 | 0.00025 |
| TMEM92-AS1 | 3.1205086 | 0.000255 |
| LINC01814 | 2.7427535 | 0.00026 |
| NQO2-AS1 | -2.5726702 | 0.000264 |
| SMG1P5 | 0.7383004 | 0.000275 |
| SNHG11 | -1.6082576 | 0.000288 |
| PARTICL | 1.5815737 | 0.000295 |
| SLC16A1-AS1 | -2.6069038 | 0.000308 |
| KDM2B-DT | -4.8244402 | 0.000312 |
| LRP1-AS | 1.9325607 | 0.000316 |
| SZT2-AS1 | 1.2727035 | 0.000321 |
| SLFNL1-AS1 | -1.8745637 | 0.00033 |
| MANEA-DT | -1.8649214 | 0.000331 |
| LINC02816 | 3.6982267 | 0.000334 |
| ELOA-AS1 | -1.3760447 | 0.000335 |
| MAGI2-AS3 | -3.6888936 | 0.000354 |
| FBXW7-AS1 | 1.3226116 | 0.000355 |
| CIRBP-AS1 | 0.886603 | 0.000391 |
| MIATNB | 0.8674932 | 0.000398 |
| PRRT3-AS1 | 1.9657652 | 0.0004 |
| LINC01238 | -2.2179077 | 0.000405 |
| TCAF2P1 | 1.4254365 | 0.000426 |
| AGAP12P | -1.9230908 | 0.000429 |
| PGM5P2 | 1.3365239 | 0.000431 |
| LINC02774 | 1.8753812 | 0.000441 |
| EHMT2-AS1 | -1.3810682 | 0.00046 |
| CHROMR | -1.1390582 | 0.000477 |
| B4GALT1-AS1 | 1.2271432 | 0.000482 |
| LINC02731 | -4.8315917 | 0.000506 |
| PAXBP1-AS1 | 1.0756673 | 0.000529 |
| MATN1-AS1 | -2.1664991 | 0.000535 |
| CLCA4-AS1 | 1.4009769 | 0.000538 |
| DSTNP2 | 1.4922311 | 0.000546 |
| ASB16-AS1 | 0.911271 | 0.000572 |
| LINC00891 | -3.6075219 | 0.000572 |
| STAG3L5P-PVRIG2P-PILRB | -0.6490593 | 0.000588 |
| FAM185BP | 1.0591669 | 0.000592 |
| LINC00265 | 1.2097396 | 0.000604 |
| SNHG19 | -1.9686099 | 0.000619 |
| NFYC-AS1 | 0.931151 | 0.00062 |
| SNHG25 | -2.6983958 | 0.000641 |
| UBE2Q1-AS1 | 0.8715964 | 0.00066 |
| LINC00954 | 1.2329376 | 0.000678 |
| LINC01569 | -2.1042309 | 0.000681 |
| RORA-AS1 | -1.9267689 | 0.000693 |
| LINC02766 | 2.2255465 | 0.000715 |
| LRRC8C-DT | -2.2550953 | 0.000733 |
| UBAC2-AS1 | -2.8572539 | 0.000808 |
| LINC01819 | 2.2674067 | 0.000857 |
| LINC01841 | 2.1160359 | 0.000866 |
| FIRRE | -1.8318116 | 0.000889 |
| EML4-AS1 | 1.5693362 | 0.000904 |
| LINC01237 | -4.0110115 | 0.000912 |
| KLHL6-AS1 | 2.3131654 | 0.000921 |
| FAM215B | 1.8736982 | 0.000926 |
| FAM66B | -3.5641842 | 0.000957 |
| ANKRD20A5P | -3.3093955 | 0.00101 |
| PSMG3-AS1 | -1.1457361 | 0.00102 |
| CERNA1 | -2.652163 | 0.00104 |
| PRKCZ-AS1 | 1.494566 | 0.00105 |
| LINC02762 | 1.9300216 | 0.00113 |
| ALOX12-AS1 | 1.0631518 | 0.00115 |
| SUGT1P3 | -2.2798633 | 0.00116 |
| VIPR1-AS1 | -2.1785506 | 0.00117 |
| DHDDS-AS1 | 1.3002082 | 0.00119 |
| NKAPP1 | -1.7067382 | 0.0012 |
| LPP-AS1 | 1.8728872 | 0.00127 |
| TTC3-AS1 | -2.4794361 | 0.00135 |
| RNFT1-DT | 1.4827807 | 0.00143 |
| SREBF2-AS1 | 1.5144515 | 0.00144 |
| LINC02413 | 2.6930362 | 0.00144 |
| A2M-AS1 | -1.8899094 | 0.00149 |
| LINC01545 | 2.0433181 | 0.00151 |
| SPATA13-AS1 | -2.5949195 | 0.00157 |
| LINC01762 | 2.3325996 | 0.00157 |
| LIMS1-AS1 | 1.8744334 | 0.00162 |
| MYLK-AS2 | -3.1525903 | 0.00164 |
| L3MBTL2-AS1 | -0.9943534 | 0.00165 |
| CARD8-AS1 | 0.6528531 | 0.00166 |
| CMAHP | 1.0105014 | 0.00167 |
| MIR4453HG | -1.080551 | 0.00167 |
| CROCCP3 | 0.9174282 | 0.00187 |
| LINC01635 | -2.3859255 | 0.00187 |
| SNHG17 | -0.7747253 | 0.00189 |
| LACTB2-AS1 | -1.9677316 | 0.00191 |
| ZNF649-AS1 | -3.2642091 | 0.00195 |
| SLC25A25-AS1 | -0.9014997 | 0.00197 |
| ULK4P1 | 2.3329905 | 0.00197 |
| RERE-AS1 | 1.9208062 | 0.002 |
| UBE2Q2P2 | -2.9465798 | 0.00212 |
| MORF4L2-AS1 | -2.0652406 | 0.00214 |
| MFSD13B | 2.7071915 | 0.00222 |
| TMEM44-AS1 | 1.3764595 | 0.00222 |
| SORD2P | -1.5597648 | 0.00223 |
| BTG3-AS1 | -2.2487288 | 0.00223 |
| ODC1-DT | -2.1386422 | 0.00224 |
| HBBP1 | -3.4139423 | 0.00226 |
| LMNTD2-AS1 | -1.7171133 | 0.00229 |
| ARRDC1-AS1 | -0.9475565 | 0.00235 |
| FAM30A | -2.191698 | 0.00236 |
| LINC02709 | 1.5945007 | 0.00236 |
| NRSN2-AS1 | -2.9387966 | 0.00237 |
| DNM1P46 | -2.428825 | 0.0024 |
| LINC02804 | -3.0111446 | 0.0024 |
| PPIEL | -1.3310837 | 0.00257 |
| CAHM | 1.2530632 | 0.00257 |
| MALINC1 | -2.3429757 | 0.00264 |
| SLC22A20P | 1.7486935 | 0.0029 |
| RPL23AP7 | -1.0029788 | 0.00292 |
| SIGLEC17P | -1.5116449 | 0.00297 |
| KDM4A-AS1 | 0.9983715 | 0.00306 |
| MYLK-AS1 | -2.2735307 | 0.00307 |
| MEF2C-AS2 | -1.5424708 | 0.00324 |
| DAPK1-IT1 | 2.6969146 | 0.0034 |
| OXCT1-AS1 | -2.2975964 | 0.00384 |
| PEF1-AS1 | 1.3702518 | 0.00397 |
| FAM66C | -1.7826784 | 0.00419 |
| PKN2-AS1 | 1.6732032 | 0.00434 |
| RPS10P7 | -2.1548157 | 0.00456 |
| MIR181A2HG | 1.6482335 | 0.00456 |
| EMC3-AS1 | 1.0263448 | 0.0047 |
| LINC00957 | -1.099672 | 0.00471 |
| STAG3L5P | 0.694829 | 0.00479 |
| DLGAP1-AS1 | 0.6702908 | 0.00485 |
| MIR155HG | -2.4237747 | 0.00489 |
| RRN3P3 | 0.7437775 | 0.00503 |
| LINC01888 | 1.1779262 | 0.0051 |
| FRY-AS1 | 1.3037205 | 0.00523 |
| TMEM254-AS1 | -1.9503143 | 0.00532 |
| VIM-AS1 | 0.9884798 | 0.00536 |
| LINC00896 | -2.5789726 | 0.00539 |
| GLIDR | -1.994626 | 0.00553 |
| TSPOAP1-AS1 | 0.9888414 | 0.00556 |
| LINC02765 | 1.726457 | 0.00562 |
| MADD-AS1 | 1.0620193 | 0.00563 |
| ZNF790-AS1 | -2.3273782 | 0.00569 |
| A2MP1 | -2.0717764 | 0.00589 |
| PRMT5-AS1 | 0.9060196 | 0.00598 |
| RRP7BP | -0.9669784 | 0.00609 |
| ERICH6-AS1 | -1.8731296 | 0.00618 |
| PARGP1 | -0.9613533 | 0.00619 |
| SHLD2P1 | -1.7651493 | 0.00648 |
| ZBTB20-AS4 | 1.5713104 | 0.00662 |
| TM4SF19-AS1 | -2.036043 | 0.00681 |
| UFL1-AS1 | -1.6301742 | 0.00689 |
| LINC00482 | 1.9271328 | 0.00703 |
| STK24-AS1 | 1.4400785 | 0.0071 |
| DENND10P1 | 0.7512724 | 0.00733 |
| LINC01970 | 1.972009 | 0.0074 |
| INE2 | -1.6574508 | 0.00741 |
| KIR3DX1 | -1.5433509 | 0.00748 |
| LINC01465 | 1.7095109 | 0.00748 |
| GLYCTK-AS1 | 1.2411512 | 0.00748 |
| FAM223A | 1.5782581 | 0.00801 |
| HCG25 | -0.7584881 | 0.00806 |
| SEC22B4P | 0.8192151 | 0.00844 |
| RTCA-AS1 | -1.5021323 | 0.00851 |
| LINC02354 | 1.5899247 | 0.00878 |
| PPP1R35-AS1 | 0.5888069 | 0.00885 |
| THAP9-AS1 | 1.2192859 | 0.00914 |
| ANP32AP1 | 1.3854467 | 0.00917 |
| LINC01126 | 0.966442 | 0.00917 |
| ZNF37BP | -0.4916362 | 0.00923 |
| PI4KAP2 | -0.7015055 | 0.00928 |
| GOLGA6L5P | 1.4669688 | 0.00938 |
| RELA-DT | 0.8888205 | 0.00939 |
| BCDIN3D-AS1 | -1.02452 | 0.0094 |
| LINC01786 | -1.5071487 | 0.00942 |
| NORAD | 0.6912261 | 0.0096 |
| LINC01215 | -1.256054 | 0.00966 |
| TMEM18-DT | -1.7990986 | 0.00979 |
| NAPA-AS1 | 1.2040483 | 0.0102 |
| LINC00339 | -0.7119815 | 0.0103 |
| PACERR | -2.0182084 | 0.0106 |
| GRK5-IT1 | 1.1770664 | 0.0107 |
| LINC01359 | 1.7427688 | 0.011 |
| CCDC162P | 1.7694383 | 0.0113 |
| RFPL1S | -2.3536295 | 0.0113 |
| PMS2CL | 0.5664591 | 0.0114 |
| ZNF702P | -2.0585498 | 0.0114 |
| LINC02285 | 1.2750658 | 0.0115 |
| ITFG2-AS1 | -1.2527932 | 0.0116 |
| LIX1L-AS1 | -0.7072993 | 0.0117 |
| SVIL2P | -1.4069199 | 0.0121 |
| AP4B1-AS1 | 1.0217624 | 0.0123 |
| PPP3CB-AS1 | -0.6213775 | 0.014 |
| FLNB-AS1 | 1.1534539 | 0.014 |
| TTC39C-AS1 | -2.3483521 | 0.014 |
| CIDECP1 | 0.6411424 | 0.0142 |
| SPATA3-AS1 | -2.451638 | 0.0142 |
| SUZ12P1 | -0.5713516 | 0.0142 |
| EP400P1 | -0.9500991 | 0.0144 |
| ZNF271P | -0.5960273 | 0.0146 |
| ZNF710-AS1 | 0.6797144 | 0.0149 |
| HLA-F-AS1 | 0.877667 | 0.015 |
| FAM223B | 1.5301712 | 0.0152 |
| ZNF451-AS1 | 1.5461134 | 0.0153 |
| MRPS9-AS1 | -1.6604339 | 0.0153 |
| SMG7-AS1 | 1.5382754 | 0.0153 |
| LINC00664 | 2.8524583 | 0.0153 |
| AATBC | -0.9428831 | 0.0154 |
| DLEU2 | 0.6763423 | 0.0158 |
| LINC00239 | 1.8390773 | 0.016 |
| LINC00677 | 1.5269075 | 0.016 |
| NSMCE1-DT | 1.8423021 | 0.0162 |
| PRORSD1P | -0.8807113 | 0.0163 |
| LINC00656 | 1.4086265 | 0.0165 |
| LINC00877 | 1.348028 | 0.0168 |
| TXNDC12-AS1 | 1.3117691 | 0.0169 |
| IL21R-AS1 | -1.1066103 | 0.017 |
| GUSBP5 | -2.3691415 | 0.0171 |
| LINC02256 | 1.1088117 | 0.0171 |
| LINC00652 | -1.5198587 | 0.0172 |
| METTL21EP | -2.3695786 | 0.018 |
| DDX11L1 | -1.0195233 | 0.0182 |
| GABPB1-AS1 | 0.632295 | 0.0182 |
| SRP54-AS1 | 0.8640769 | 0.0188 |
| HSD17B7P2 | -1.2783339 | 0.0189 |
| SPAG5-AS1 | 0.7319838 | 0.0189 |
| ZFAT-AS1 | 1.1192568 | 0.0201 |
| NDUFA6-DT | -0.8780721 | 0.0206 |
| LINC02610 | -1.9914224 | 0.0207 |
| LINC01353 | 1.4581043 | 0.0209 |
| RBM26-AS1 | -2.0447457 | 0.0211 |
| TAB3-AS1 | 1.6421498 | 0.0211 |
| ZNF571-AS1 | -1.6242999 | 0.0211 |
| CFAP58-DT | 1.109227 | 0.0216 |
| ERVH48-1 | 1.220764 | 0.0221 |
| ZNF561-AS1 | -1.3278841 | 0.0221 |
| PINK1-AS | -0.8005504 | 0.0222 |
| MST1L | -1.1462317 | 0.0222 |
| IFNG-AS1 | -1.9061495 | 0.0223 |
| ASMTL-AS1 | 0.7304583 | 0.0227 |
| EZR-AS1 | -0.8265838 | 0.0246 |
| MCPH1-AS1 | -1.3898915 | 0.025 |
| LINC02009 | 1.3465054 | 0.0253 |
| PYCARD-AS1 | 0.4953157 | 0.0255 |
| CTBP1-DT | -0.6319136 | 0.0258 |
| ANO7L1 | -1.039875 | 0.0271 |
| TMEM252-DT | 1.4033082 | 0.0277 |
| LINC00662 | 1.3740064 | 0.0291 |
| LINC02432 | -1.2250321 | 0.0293 |
| LINC01145 | -0.6020069 | 0.0299 |
| MED14OS | 0.9647934 | 0.0304 |
| LMO7-AS1 | -1.4517641 | 0.0306 |
| OGFR-AS1 | 0.8644175 | 0.0308 |
| ITFG1-AS1 | 0.7914868 | 0.031 |
| LINC01765 | -1.8416586 | 0.033 |
| MRPL20-AS1 | -0.5242807 | 0.0331 |
| WASH5P | 0.7927508 | 0.0331 |
| PMS2P3 | -0.6442336 | 0.0332 |
| PLCG1-AS1 | -0.572245 | 0.0339 |
| GMDS-DT | 0.641961 | 0.0344 |
| TPT1-AS1 | 0.8034136 | 0.037 |
| DCST1-AS1 | 1.0813257 | 0.0387 |
| TH2LCRR | -0.9908387 | 0.0388 |
| SOD2-OT1 | 1.3915052 | 0.0392 |
| LINC02352 | 0.896046 | 0.0394 |
| PAXIP1-AS2 | -0.7600894 | 0.04 |
| KIF9-AS1 | 0.7501709 | 0.0402 |
| OTUD6B-AS1 | -0.7582905 | 0.0406 |
| MHENCR | -0.6339502 | 0.0408 |
| LRRC37A4P | -0.4963609 | 0.041 |
| IL20RB-AS1 | 0.5695605 | 0.0412 |
| SYS1-DBNDD2 | -0.6682956 | 0.0412 |
| ATP6V1G2-DDX39B | 0.3906127 | 0.0414 |
| VPS13A-AS1 | -1.3111314 | 0.0416 |
| DCTN1-AS1 | 1.2350569 | 0.0424 |
| ZBED5-AS1 | -0.8376389 | 0.0427 |
| ZNF788P | 0.9903113 | 0.0428 |
| DIRC3 | -1.4523476 | 0.0434 |
| FAM87A | 1.3973635 | 0.0461 |
| EIF1AX-AS1 | -1.2653288 | 0.0468 |
| SGO1-AS1 | -1.4203075 | 0.048 |
| DHRS4L1 | -1.2618386 | 0.0488 |
| DOC2GP | -1.4356874 | 0.0488 |
| LINC00863 | -0.6540589 | 0.0495 |
| LINC02569 | 1.360908 | 0.0497 |
