# Supplementary material for: Identification of immune-related lncRNA in sepsis by construction of ceRNA network and integrating bioinformatic analysis
Source: BMC Genomics. 2023 Aug 24;24:484. doi: 10.1186/s12864-023-09535-7 (PMC10464037; doi:10.1186/s12864-023-09535-7)
Supplement: Supplementary file 4 — Additional file 4: Supplementary Figure S1. Validation of the DElncRNAs in GSE217700 dataset. (A) The volcano plot of DElncRNAs in GSE217700 dataset. (B) Box plots of LINC00265 and PRKCQAS1 expression between sepsis patients and normal controls in GSE217700. (C) Venn diagram of 6 hub lncRNAs and GSE217700; red ellipse indicates 6 candidate lncRNAs, blue ellipse indicates DElncRNAs of GSE217700; overlap intersection indicates common shared DElncRNAs [file 12864_2023_9535_MOESM4_ESM.docx]

**
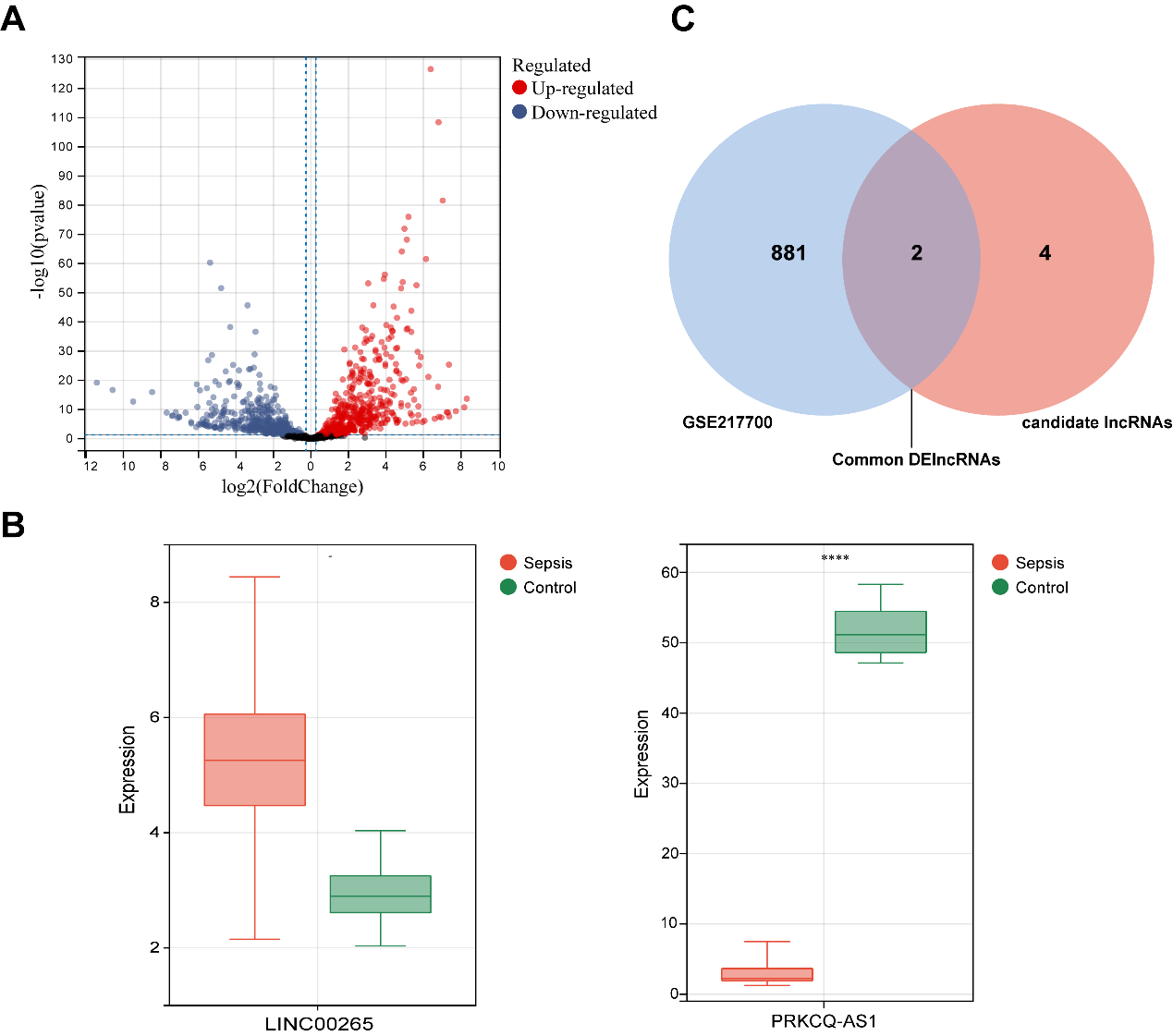
**

**Supplementary Figure S1. Validation of the DElncRNAs in GSE217700 dataset.** (A) The volcano plot of DElncRNAs in GSE217700 dataset. (B) Box plots of LINC00265 and PRKCQAS1 expression between sepsis patients and normal controls in GSE217700. (C) Venn diagram of 6 hub lncRNAs and GSE217700; red ellipse indicates 6 candidate lncRNAs, blue ellipse indicates DElncRNAs of GSE217700; overlap intersection indicates common shared DElncRNAs.
