# Supplementary material for: Identification of immune-related lncRNA in sepsis by construction of ceRNA network and integrating bioinformatic analysis
Source: BMC Genomics. 2023 Aug 24;24:484. doi: 10.1186/s12864-023-09535-7 (PMC10464037; doi:10.1186/s12864-023-09535-7)
Supplement: Supplementary file 5 — Additional file 5: Supplementary Figure S2. Machine learning in the identification of hub genes. (A, B) Identified optimal IRDEGs by using LASSO regression and SVM algorithms. (C) The intersection of candidate hub genes between LASSO, SVM and PPI module analysis [file 12864_2023_9535_MOESM5_ESM.docx]

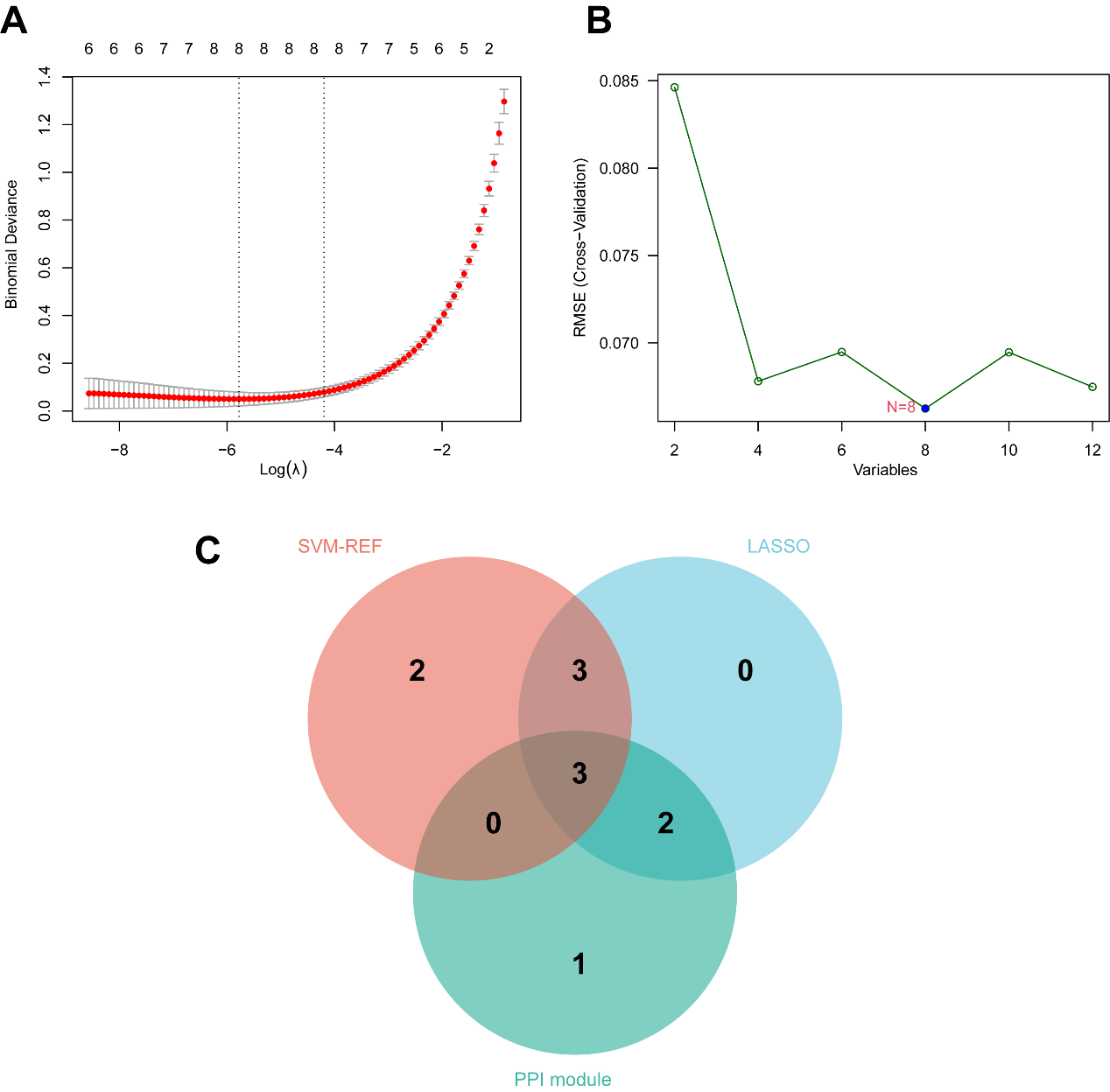


Supplementary Figure S2. Machine learning in the identification of hub genes. (A, B) Identified optimal IRDEGs by using LASSO regression and SVM algorithms. (C) The intersection of candidate hub genes between LASSO, SVM and PPI module analysis.
